# Exploring the prevalence of childhood adversity among university students in the United Kingdom: A systematic review and meta-analysis

**DOI:** 10.1371/journal.pone.0308038

**Published:** 2024-08-28

**Authors:** Jackie Hamilton, Alice Welham, Gareth Morgan, Christopher Jones

**Affiliations:** 1 Department of Neuroscience, Psychology and Behaviour, University of Leicester, Leicester, United Kingdom; 2 Depatment of Psychology, University of Birmingham, Birmingham, United Kingdom; Universitat der Bundeswehr München: Universitat der Bundeswehr Munchen, GERMANY

## Abstract

**Objectives:**

The focus of this review was to systematically review and meta-analyse the prevalence of ACEs among university students in the UK.

**Method:**

The systematic searching of six electronic databases (conducted February 2024) identified ten relevant articles (peer-reviewed articles of a quantitative nature that included ACE prevalence). PROSPERO reference: CRD42022364799.

**Results:**

Pooled prevalence for number of ACEs endured was 55.4% (95% CI: 32.4% - 78.4%; I^2^ > 99.5%) for one or more, and 31.6% (7.5% - 55.6%; I^2^ > 99.5%) for three or more. Pooled prevalence was: 15.9% (7.0% - 24.7%; I^2^ > 94.5%) for physical abuse; 27.0% (18.1% - 35.9%; I^2^ > 94.5%) for emotional abuse; 12.1% (5.2% - 19.0%; I^2^ > 94.5%) for sexual abuse; 8.4% (1.7% - 15.1%; I^2^ > 95.4%) for physical neglect, and 30.0% (21.5% - 38.5%; I^2^ > 95.4%) for emotional neglect. Pooled prevalence for household dysfunction categories were: 34.4% (22.8% - 46.0%) for parental separation; 18.4% (10.1% - 26.8%) for domestic violence; 35.2% (23.6% - 46.8%) for mental health difficulties; 21.4% (12.9% - 29.9%) for substance use; and 5.7% (2.3% - 9.1%) for incarceration (I^2^ > 88.8% for all household dysfunction items). Significant heterogeneity was observed between studies for most categories of adversity, and it was not possible to explain/reduce this variance by removing small numbers of influential/discrepant studies. Further analyses suggested potential influences of measurement tool used, country of data collection, and age and sex of participants.

**Conclusion:**

Results demonstrate considerable, largely unaccounted-for, heterogeneity in estimates of the prevalence of ACEs, impeding confidence in any summary statistics. Conclusions must be tentative due to analyses being underpowered given small numbers of papers, as well as potential confounds, meaning results may not be truly representative. However, results do suggest high prevalence rates which warrant further investigation, with appropriate support offered to students.

## Introduction

The concept of childhood adversity has received international attention, partly stemming from a large epidemiological study conducted in the US [1]. This study focused on the prevalence of adverse childhood experiences (ACEs), finding that thirty-five percent of the US population (*N* = 9,508) reported three or more types of ACEs. Following this, further research exploring the prevalence of ACEs has been conducted worldwide, with a study in the UK demonstrating that 47% of people experienced at least one ACE and 9% of the population report experiencing four or more ACEs [2].

Due to considerable variability in the literature, it has proved difficult to find a universal definition of childhood adversity, despite the substantial body of research examining childhood adversity. McLaughlin and colleagues refer to childhood adversity as environmental experiences that require adaptation by a child, and that represent a deviation from what is expected [3]. They argue that for experiences to be considered as adversity, the threat or deprivation must be chronic (e.g., ongoing emotional abuse from caregivers, ongoing separation from caregivers), or include single events that are severe enough to have an emotional, cognitive, or neurobiology impact on the child (e.g. sexual abuse, [3]). This is broadly congruent with the definition provided by the American Psychological Association [4], who define trauma as events that result in significant threat to the safety of an individual or their loved ones/friends.

Adverse childhood experiences are thought to encompass a wide range of early traumatic events or chronic stressors (such as sexual abuse, physical abuse, emotional abuse, neglect, as well as areas of household dysfunction (such as alcohol and substance abuse, parental separation, domestic violence, parental mental health difficulties), deprivation, bullying, and peer, community, and collective violence [3, 5]).

Adverse childhood experiences are one of the strongest predictors of poor health and social outcomes during adulthood [6], and is thought to impact a child’s physical, behavioural, and cognitive development [7, 8]. Research suggests that the impact of ACEs exposure may be greatest during very early and early childhood, when it may coincide with vital childhood developmental timeframes [9]. Research demonstrates that ACEs can be associated with a range of later physical health difficulties (including heart disease, diabetes, asthma, cancer, and other chronic conditions; [1, 6, 7, 10]), and elevated distress (including sleep difficulties, low mood, anxiety, post-traumatic stress reactions, difficulties with substance use; and difficulties with social functioning; [7, 11–14]). It has been argued that it is advantageous to recognise and offer support for trauma as early as possible, as mental health needs may become harder to manage if intervention is sought later in life [15, 16].

Conversely, research also highlights that not all children who are exposed to ACEs experience heightened distress [17, 18]. This is thought to be linked to the presence of protective factors that may mediate the relationship between ACEs and distress, as they nurture so-called ‘resilience’ and diminish the potential negative impacts of ACEs [2, 19]. These factors include having a strong sense of purpose in life, a high education level, good levels of social support, and being male; factors which are sometimes considered to be associated with ‘resilience’ and ‘recovery from’ adversity [17, 20, 21]. However, others have recognised that such factors can be linked to differing levels of privilege and access to social and material resources; conceptualising resilience as a character trait of an individual is problematic as it depoliticizes resilience from the wider socio-political context [22, 23].

Childhood trauma is thought to have a negative impact on an individual’s academic performance, suggesting that individuals who experience childhood adversity may be less likely to progress into higher education [24]. Thus, it may be plausible to propose that the prevalence of ACEs may be lower among university students compared to the general population; however, some studies demonstrate a high prevalence among university students in the UK (79% - 84%; [25, 26]).

As pointed out by Davies et al. [25], most prevalence studies have, justifiably, focused on general populations or populations of people accessing mental health services. However, it has been argued that university students form a unique population who are going through an important life transition, whereby the impact of ACEs may influence their social and/or academic performance [25–27].

The transition to university involves moving away from family and friends, navigating a new environment, academic pressures, financial pressures, new social relationships, and making decisions about risky health behaviours [20, 28–30]. These factors may impact on an individual’s wellbeing and are likely to result in heightened distress for most students [31]. The mental health of university students has received a lot of attention over the recent years and is a major health concern [32–34], as over a third of students report problems with low mood and/or anxiety within their first year of university [35]. The transition to university also occurs alongside the challenges of transitioning to adulthood, coinciding with the peak risk of being assigned a mental health diagnosis before the age of 24 years old [36].

The transition to university may be a challenging time for most students; however, individuals who have experienced ACEs may find this adjustment even more difficult [37]. Individuals who experience ACEs tend to report greater distress [38], and research has found an association with high-risk behaviours, physical diseases, and poorer academic performance [24, 39, 40]. Sheldon et al. [41] suggest that ACEs are an important risk factor that could enable universities and healthcare services to identify and provide support to those in need.

Some researchers argue that students who have experienced ACEs are an important but often overlooked subgroup [42]; therefore, it is important that this population are given distinct research attention. Questions remain about both the prevalence of ACEs and the impact for students. Internationally, this has begun to be explored; Fu et al. [42] conducted a systematic review (of five relevant databases) and meta-analysis which explored the prevalence of childhood maltreatment among university students in China. They included ACEs that related to childhood maltreatment only (physical abuse, emotional abuse, sexual abuse, physical neglect, and emotional neglect) which were measured using validated measurement tools. The pooled prevalence results indicated that 64.7% of university students experienced childhood maltreatment; however, high levels of heterogeneity were observed for the overall estimate and for all subtypes of childhood maltreatment, thus, results must be interpreted with caution. Additionally, Sheldon et al. [41] conducted a systematic review (of four relevant databases) and meta-analysis which explored risk factors for distress among university students in the UK. This review focused on undergraduate students only, and the inclusion criteria specified that studies needed to contain longitudinal observations of cohorts or case-control samples. Although the study found that ACEs were predictive of suicidality, due to the focus being on risk factors, the prevalence of ACEs among this population were not explored. Similar findings were reported as above regarding high levels of heterogeneity between studies and caution associated when interpreting these findings.

To the best of our knowledge, there is no systematic review or meta-analysis on the prevalence of ACEs among university students in the UK. Quantifying the prevalence of ACE exposure may contribute to understanding the needs of this unique population to inform better policies, support, and services at universities. Thus, this review aims to systematically review and meta-analyse the prevalence of ACEs among university students in the UK. To assess any potential sources of heterogeneity, possible confounding factors were considered, including the type of participants, measurement tool used, number of ACEs measured in study, and the country of study. The potential impact of the following moderators were also included in meta-regression analyses: age, sex, and risk of bias score.

For consistency with the wider literature on ACEs and ease of understanding, ACEs were considered in the categories that have previously been defined in the literature [6]: childhood abuse (consisting of physical abuse [PA], emotional abuse [EA] and sexual abuse [SA]), childhood neglect (consisting of physical neglect [PN] and emotional neglect [EN]), and household dysfunction (consisting of parental separation [PS], domestic violence [DV], mental health problems [MHP], substance abuse [Sub], incarceration [Inc]). Any additional ACEs which were reported but did not fit in these categories were also explored (such as peer-victimisation and deprivation).

## Method

### Search strategy and sources

This review was written in line with the Preferred Reporting Items for Systematic Reviews and Meta Analyses (PRISMA) guidelines [43]. This study is registered on the PROSPERO database (CRD42022364799).

A systematic literature search was conducted between 26^th^ February– 8^th^ March 2024 using the following six databases: Allied and Complementary Medicine (AMED); British Education Index (BEI); Cumulative Index to Nursing and Allied Health Literature (CINAHL); Education Resources Information Center (ERIC); PubMed; and PsycINFO. These databases provide a comprehensive search of research related to psychology, education, health, and medicine. Reference lists of identified articles and relevant review articles were examined to ensure all suitable articles were included.

Search terms relating to the areas of interest for this review–adversity (e.g. ‘trauma’, ‘abuse’), university students (e.g. ‘student’, ‘university’), and location (e.g. ‘United Kingdom’, ‘Britain’)–were generated and used in the literature search (S1 Appendix). Search terms were used on all databases to search titles, abstracts and keywords, excluding PsycINFO (due to circa 40,000+ results being identified, therefore, following consultation with a research services specialist from the library, results were narrowed by searching for the ‘location’ search term in author affiliation and location only). The selection of search terms was guided by previous systematic reviews in similar areas [42, 44, 45] and consultation with librarians.

The initial literature search was done by one reviewer (JH), who then also retrieved full-text articles, and two reviewers screened these full-text articles (JH and AW). Any conflicts over inclusion were resolved through discussion between JH and AW. Date were extracted by JH and checked over by AW.

### Selection criteria

#### Search limits

Search limits include papers published in peer-reviewed journals, papers published in English language, and papers published since the millennium (2000–2022).

#### Inclusion criteria (all criteria must be met for inclusion)

Inclusion criteria include university student sample (or identifiable sub-sample of university students, with separate data reported), location of student sample in United Kingdom only, assessment/reporting of exposure to childhood adversity (adverse childhood experience before the age of 18 years old), quantitative methodology, and prevalence of adverse childhood experience data available (or directly calculable from the paper).

#### Exclusion criteria

Exclusion criteria include non-peer reviewed journals, letters to the editor, proceedings, theses, qualitative data, non-university sample, non-UK location, prevalence of overall adverse experiences (where separate childhood adversity data was not available).

### Article selection summary

In total, 6376 articles were identified across the six databases. Duplicates were removed (n = 2109), and a further 4204 papers were removed after screening of abstracts revealed papers did not meet inclusion criteria. The remaining 63 articles were read in full. Two additional articles were identified through reviewing the reference lists for other relevant research. Of these 65 articles, 13 were found to report prevalence data on ACEs among university students in the UK. There were five instances where articles met the inclusion criteria; however, they included the same data set as another study included in the review. They were excluded at this point if they did not add any additional ACE prevalence data above and beyond the article already included [46–51].

Where prevalence data was not available in the published article, study authors were contacted for further information. Four papers provided only mean and standard deviation data which was not convertible to prevalence (%) data [14, 52–54] and one paper included the total ACE score into regression models, but no details regarding individual ACEs was available [55]. These papers were excluded at this point.

**Fig 1:**
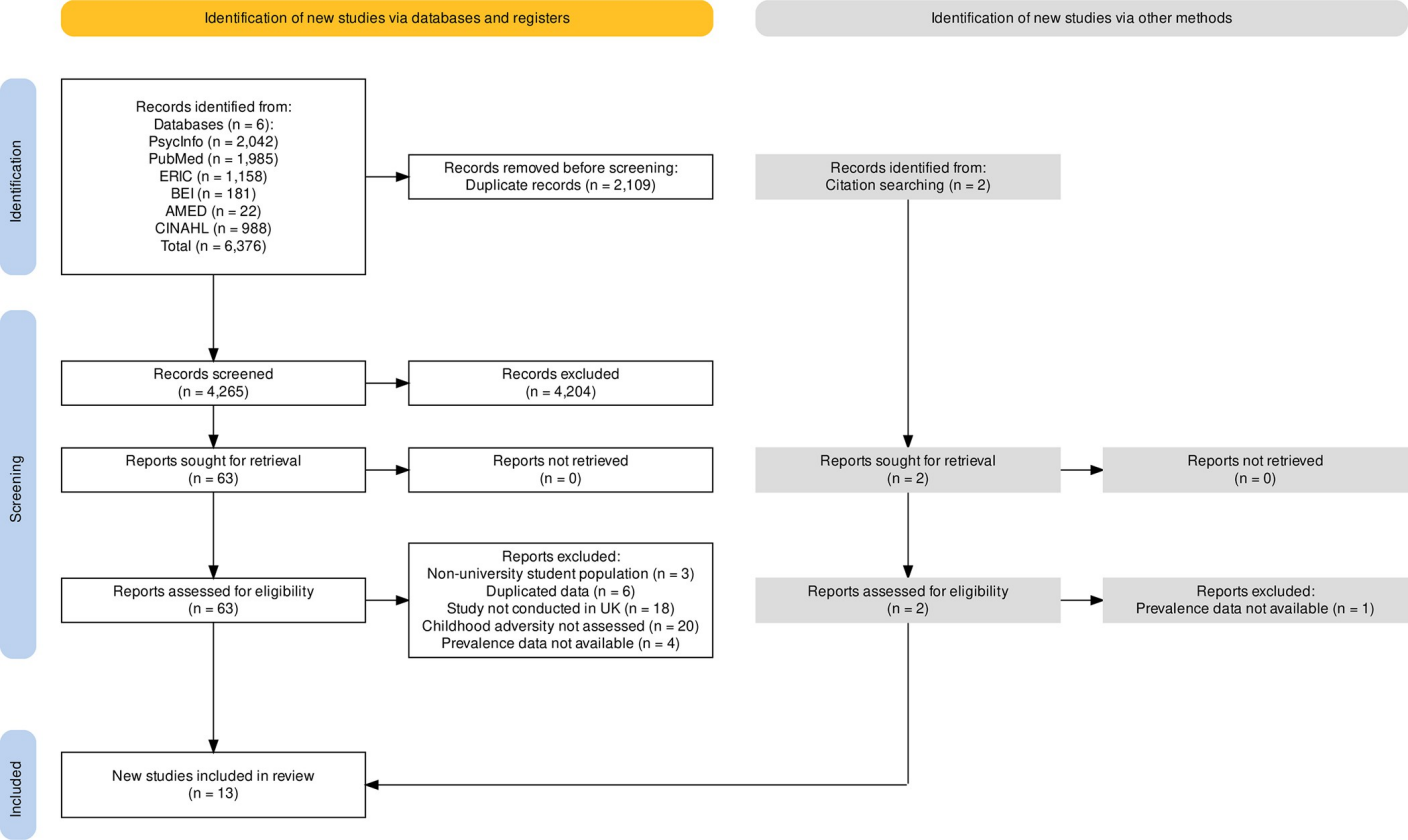
A PRISMA flow diagram of the systematic search procedure (Page et al., 2021). Note: AMED = Allied and Complementary Medicine; BEI = British Education Index; CINAHL = Cumulative Index to Nursing and Allied Health Literature; ERIC; Education Resources Information Center.

Where two or more papers reported prevalence data on any particular category of ACE, meta-analysis was carried out [56]. Only one paper reported the prevalence rate of peer-victimisation [57], one paper reported on childhood threat [58], and one paper provided the prevalence rate for deprivation [59]; thus, it was not possible to meta-analyse these categories of ACEs. Ten papers were consequently included in the meta-analyses [25, 26, 60–67] exploring ten types of adverse childhood experiences (PA [n = 7]; EA [n = 8]; SA [n = 9]; PN [n = 6]; EN [n = 5]; PS [n = 3]; DV [n = 5]; MHP [n = 4]; Sub [n = 4]; Inc [n = 2]), as well as the prevalence of one or more ACE (n = 5) and three or more ACEs (n = 4). A PRISMA flowchart summarises the article selection process (Fig 1).

### Data extraction

Information was collated regarding year of publication, location of university students from whom data were collected, aims of the study, sample size, population type, mean age, sex, ethnicity, assessment tool(s), and prevalence data.

### Quality/bias appraisal tool

Studies were rated on risk-of-bias criteria (Table A in S2 Appendix) relating specifically to prevalence estimates for the purposes of the current meta-analyses. Criteria were based on (i) sample/recruitment, (ii) consistency of definition of ACE with agreed standard definition (Table B in S2 Appendix), and (iii) quality of ACE assessment tool. Criteria were bespoke for the current review, but informed by the Cochrane risk-of-bias tool [68] and previous similarly-informed tools for pooled prevalence meta-analyses [69]. Risk-of-bias appraisal was completed for each paper included in the meta-analyses, and for each category of ACE that was being meta-analysed. These criteria specifically focus on the individual ACE and key threats to validity for this current review; therefore, scores should not be taken as ‘quality’ ratings of the papers in general. All studies included in the meta-analyses were rated by the first author (JH) using the criteria, and these were used in the analysis. However, agreement with blind ratings from a second rater (AW) was also assessed for the RoB ratings for (i) sample/recruitment, as well as ratings for (ii) definition of ACE, (iii) ACE assessment tool, and overall ROB rating for sexual abuse (the category involving the most papers). Weighted kappa indicated perfect agreement on the assessment tool (1.0), and was substantial for overall ROB rating (.68) and definition of ACE (.64). For the sample/recruitment measure, percentage agreement was 80% (lack of variability in the primary rater’s measurements on this criterion precluded calculation of weighted kappa).

### Meta-analysis of prevalence

Pooled prevalence meta-analyses were conducted for each ACE where two or more papers reported prevalence data. Ten ACEs were covered within these papers and are discussed and presented in the three over-arching categories that have previously been defined in the literature: childhood abuse (PA, EA, SA), childhood neglect (PN and EN), and household dysfunction (PS, DV, MHP, Sub, Inc), as well as overall prevalence of adversity (one or more ACEs and three or more ACEs; categories based on available data within papers).

Random-effects models were used to allow for potential variability between studies, using the generic inverse variance method [69, 70]. The DerSimonian and Laird method (DL; [71]) was used where initial Q-Q plots did not denote deviations from normality for prevalence estimates (one or more ACEs; three or more ACEs; EN, PS, DV, MHP, Inc); however, for those that did show deviations from normality (PA, EA, SA, PN, Sub), the restricted maximum likelihood estimator (REML) was used instead due to its robustness with violations of normality [72]. Due to low *n* of studies within the meta-analyses (and so caution in concluding adherence to normality assumptions), meta-analyses were also run using the REML method, but minimal differences were found. Heterogeneity was explored using the I^2^ statistic and Cochran’s chi-squared test (Cochran’s Q), whereby values of I^2^ > 75% indicated considerable heterogeneity [73].

A leave-one-out analysis was used to explore the influence of individual studies on the results, and publication bias and small study effects were examined through the use of funnel plots (with caution employed when fewer than ten studies were included in the meta-analyses; [56, 74]). A quality effects model was also utilised with adjusted weightings according to studies’ overall risk of bias ratings.

To identify possible sources of heterogeneity, subgroup analyses were used to assess the possible impact of the following categorical variables: type of participants (all university students vs psychology students only), measurement tool used to assess ACE, number of ACEs measured in study (fewer than ten vs ten or more) and country of study. Meta-regression analyses were used to explore the potential impact of the following continuous/ordinal variables: mean age of participants, proportion of females in sample, and overall quality/risk of bias score. Subgroup analyses are considered a core component of meta-analyses, particularly when heterogeneity is present, as recommended by the Cochrane Handbook [56]. However, Pigott [75] highlights that a problem of subgroup analyses is that they have low power, with Cuijpers, Griffin, and Furukawa [76] reporting that in comparison to an ‘average’ meta-analysis, a subgroup analysis requires 3–4 times the number of studies to have sufficient power, and this number of studies increases with higher heterogeneity and unequal numbers of studies in the subgroups. Therefore, results are interpreted with caution. Meta-analyses were conducted in R (version 4.0.4), using the Metafor package, version 3.6.2.

## Results

### Study characteristics

The characteristics of studies included in the meta-analyses are presented in Table 1. The ten articles were published between 2001 and 2022; the majority were conducted in England (*n* = 5), followed by Northern Ireland (*n* = 3), Wales (*n* = 1), and Scotland (*n* = 1). Most studies collected data from one university site only.

**Table 1:** Selected study characteristics.

Author, Year of publication	Country of Study	Sample size, n	Population	Number of universities sampled	Mean age, years, SD (Range)	Sex (Female %)	Ethnicity (White British %)	Childhood adversity measure
Davies, Read & Shevlin, 2022	England	858	First year (Level 4) students	1	27.7 ± 13.5 (Range: unknown)	69.3%	28.9%	ACE scale and CATS
Gracie et al., 2007	England	228	University students	1	28.9 ± 8.7 (Range: unknown)	70.6%	81.6%	TLEQ and CTQ
Ireland, Alderson & Ireland, 2015	England	198	University students	1	20.2 ± 2.4 (Range: unknown)	72.7%	78%	CASE
Lagdon et al., 2021	Northern Ireland	640	University students	1	Maltreated group 22.0 (Range: unknown) Non-maltreated group 21.0 (Range: unknown)	76.5%	NR	Questions developed by Christoffersen et al., 2013
Martin-Denham & Donaghue, 2022	England	156	University students	Multiple*	38.0 ± 1.77 (Range: 19–57)	NR	NR	ACE scale
McGavock & Spratt, 2017	Northern Ireland	765	Undergraduate students	1	20.7 (Range: 18–54)	72.7%	NR	ACE scale and a question regarding peer-perpetrated violence
Moulton et al., 2015	Scotland	142	Psychology undergraduate students	1	21.1 ± 4.8 (Range: 18–46)	100%	NR	CTQ
O’Neil et al., 2018	Northern Ireland	739	University students	1	20.7 ± 5.3 (Range: 18–49)	62.5%	98.2%	ACE scale and WMH-CIDI and Army STARRS
Oaksford & Frude, 2001	Wales	213	Psychology students	1	21.0 (Range: 18–41)	100%	NR	Developed own questionnaire
Worsley et al., 2018	England	1029	University students	1	19.8 ± 1.7 (Range: 17–25)	74.8%	NR	ACE scale

*Note*. SD = standard deviation; NR = not reported; ACE scale = adverse childhood experiences scale; CATS = child abuse and trauma scale; TLEQ = traumatic life events questionnaire; CTQ = childhood trauma questionnaire; CASE = checklist to assess sexual exploitation; WHM-CIDI = World Mental Health–Composite International Diagnostic Interview; Army STARRS = army study to assess risk and resilience in service members.

* = Data was collected from multiple universities, but authors did not collect data regarding which universities participants attended.

### Participant characteristics

The total number of participants was *n* = 4,968, with samples ranging from 142 [64] to 1029 [67]. Most studies included males and females in their samples; however, two papers included exclusively female samples [64, 66] and one [26] did not collect this data. The total sample was comprised of 79% females (out of 9 studies with data available), with 70% identifying as coming from a White ethnic background (from 4 papers that reported this data). The age of participants ranged from 17–57 years (weighted mean 22.7 years: all studies included participants that were attending a university in the UK). Five studies reported that their sample comprised a general sample of university students, two focused on psychology students only, one focused on undergraduate students only, and one focused on first year students only (Table 1).

### Adversity types and measures

Of the ten studies included in the meta-analyses, five reported the prevalence of one or more ACEs (3,448 participants), in which sexual abuse (SA) was the most explored ACE (nine papers; 4,740 participants), and incarceration of a household member was the least (three papers; 1,779 participants; Table 2). Several measurement tools were adopted in the selected studies, including the Adverse Childhood Experiences Scale (ACE scale), the Childhood Trauma Questionnaire (CTQ), the Child Abuse and Trauma Scale (CATS), the Traumatic Life Events Questionnaire (TLEQ), and other miscellaneous tools (Table 1).

**Table 2:** Prevalence and types of adversities measured.

Author, Year of publication	Childhood adversities measured	Total number of adversities	Prevalence % (95% CI)											
			Overall		Childhood abuse			Childhood neglect		Household dysfunction				
			1+	3+	PA	EA	SA	PN	EN	PS	DV	MHP	Sub	Inca
Davies, Read & Shevlin, 2022	EA, SA, PN, EN, PS, DV, MHP, Sub, Inc, Violence, Discrimination	12	79.3%	50.7%	NA	33.4%	18.9%	8.9%	29.0%	35.1%	23.5%	26.4%	19.5%	6.1%
Gracie et al., 2007	PA, EA, SA, PN, DV	5	NR	NR	11.8%	20.6%	NR	0.9%	NA*	NA	23.25%	NA	NA	NA
Ireland, Alderson & Ireland, 2015	SA	1	NR	NA	NA	NA	22.2%	NA	NA	NA	NA	NA	NA	NA
Lagdon et al., 2021	PA, EA, SA, neglect	4	26.1%	3.1%	16.9%	19.2%	2.2%	NA	NA	NA	NA	NA	NA	NA
Martin-Denham & Donaghue, 2022	PA, EA, SA, PN, EN, PS, DV, MHP, Sub, Inc	10	84.0%	52.0%	44.0%	56.0%	34.0%	21.0%	51.0%	47.0%	28.0%	54.0%	36.0%	10.0%
McGavock & Spratt, 2017	PA, EA, SA, PN, EN, PS, DV, MHP, Sub, Inc	10	56.0%	21.2%	11.5%	20.6%	5.9%	2.9%	20.2%	22.8%	5.7%	30.1%	16.7%	2.6%
Moulton et al., 2015	PA, EA, SA, PN, EN	5	NR	NR	10.7%	33.6%	11.4%	17.9%	37.1%	NA	NA	NA	NA	NA
O’Neil et al., 2018	PA, EA, SA, DV, MHP, Sub, neglect, insults**, physical punishment, chores***, parental criminal activity and suicidal behaviour	13	NR	NR	6.6%	14.6%	2.0%	NA	NA	NA	13.0%	32.2%	15.7%	NA
Oaksford & Frude, 2001	SA	1	NR	NA	NA	NA	13.1%	NA	NA	NA	NA	NA	NA	NA
Worsley et al., 2018	PA, EA, SA, PN, EN	5	31.8%	NR	11.7%	21.3%	2.9%	1.7%	16.7%	NA	NA	NA	NA	NA

*Note*.

* emotional abuse/neglect combined as one in paper so only used in the EA category for meta-analytic purposes

** insults received repeatedly

*** chores which were dangerous or age inappropriate; NR = not reported; NA = not applicable; CI = confidence interval; PA = physical abuse; EA = emotional abuse; SA = sexual abuse; PN = physical neglect; EN = emotional neglect; PS = parental separation; DV = domestic violence; MHP = mental health problem; Sub = substance abuse; Inc = incarceration; FSM = free school meals; For brevity’s sake, adversities which were only explored by 1 or 2 papers were not reported in this table (including peer-victimisation, deprivation, and childhood threat)

### Quality/bias appraisal

The studies were rated on risk-of-bias criteria (Table A in S2 Appendix) relating to the: (i) representativeness of sample; (ii) consistency of definition of ACE with agreed standard definition (Table B in S2 Appendix); and (iii) quality of ACE assessment tool. Criteria were scored as either unclear (0—red), poor (0—yellow), adequate (1—amber), or good (2—green). A quality effects model was utilised with adjusted weightings according to studies’ overall risk of bias ratings (calculated by dividing the total quality score by the maximum possible total of six).

Caution was taken with studies that received a quality weighting score <0.33; two studies [62, 65] scored a quality weighting of 0.17, thus, additional meta-analyses were run with these studies removed, and results are presented for comparison. The bias appraisal scores for all meta-analysed studies are included in S3 Appendix.

### Pooled prevalence meta-analyses

Pooled prevalence meta-analyses are presented below for overall prevalence of ACEs, and each category of ACE (presented under categories of ‘childhood abuse’ [consisting of PA, EA, and SA], ‘childhood neglect’ [consisting of PN and EN], and ‘household dysfunction’ [consisting of PS, DV, MHP, Sub, Inc]).

#### Overall number of ACEs

**Fig 2:**
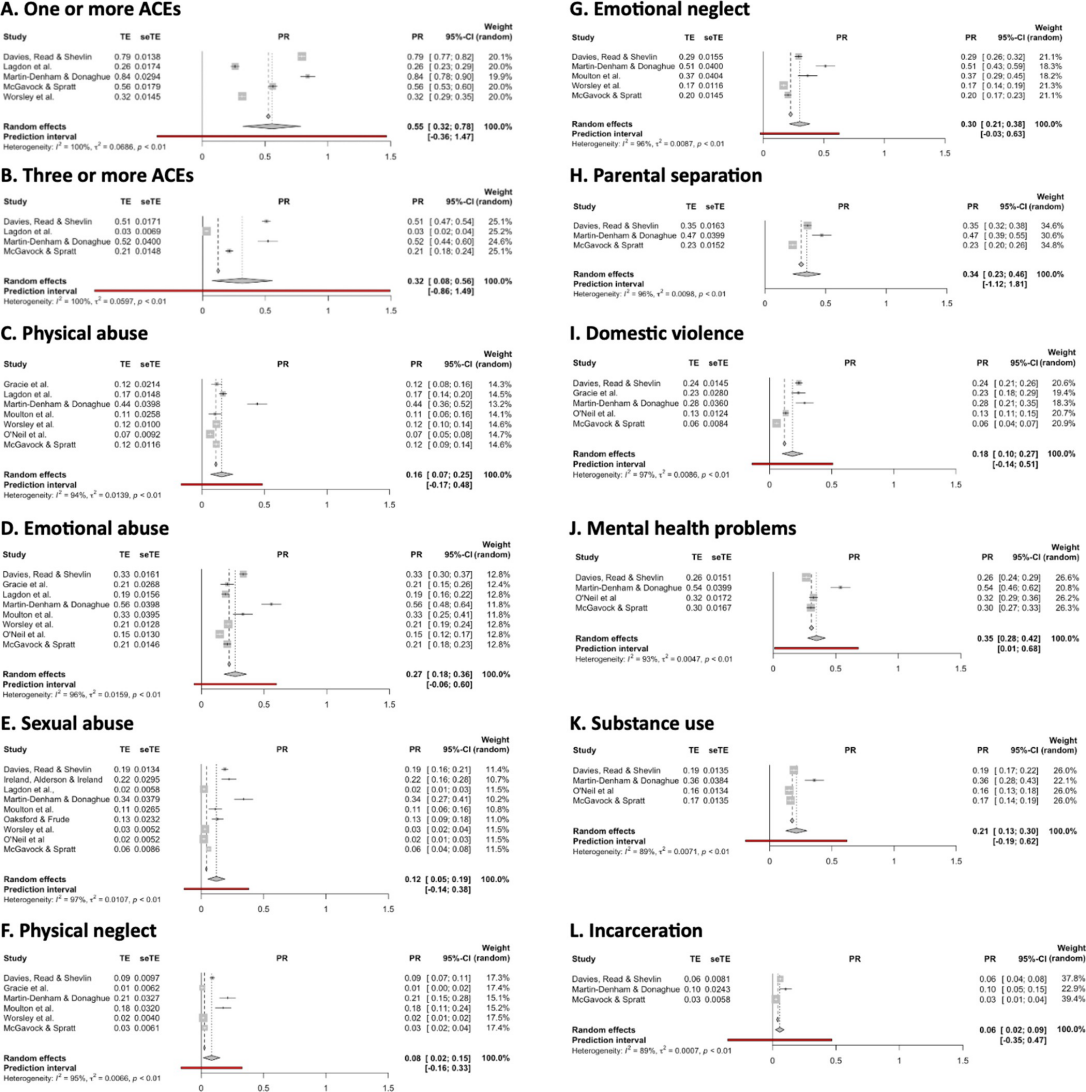
Forest plots for one or more ACEs, three or more ACEs, and all ACEs relating to childhood abuse, childhood neglect, and household dysfunction.

Random effects models suggested a weighted prevalence of 55.4% (95% CI: 32.4% - 78.4%) and 31.6% (95% CI: 7.5% - 55.6%) for one or more, and three or more, ACEs respectively (Fig 2(A) and 2(B). Significant heterogeneity was observed between studies for each of these analyses (in both cases, I^2^ > 99.5%; p < .0001). A leave-one-out analysis was conducted for each meta-analysis (S4 Appendix); however, no single study demonstrated an outsized effect on the pooled estimates. The quality effects models gave similar results, and even after removing Lagdon et al. [62] minimal differences were detected (Section A of S5 Appendix).

To explore the potential sources of heterogeneity, subgroup and meta-regression analyses were conducted (see Table 3). Results tentatively suggest the possibility that studies which use the ACE measurement tool (n = 4) reported significantly higher prevalence of one or more, and three or more ACEs. In addition, studies which measured ten or more ACEs (n = 3) resulted in a higher prevalence of one or more, and three or more ACEs. A significant negative association was found between the percentage of females in the sample and the prevalence of one or more, and three or more ACEs.

**Table 3:** Subgroup and meta-regression analyses of the prevalence of overall ACEs and individual ACEs by study characteristics.

Study characteristics	Prevalence % (95% CI)											
	Overall prevalence		Childhood abuse			Childhood neglect		Household dysfunction				
	1 or more	3 or more	PA	EA	SA	PN	EN	PS	DV	MHP	Sub	Inc
**Type of students**												
All university students	N/A	N/A	16.8 (6.4–27.2)	26.2 (16.2–36.3)	12.2 (3.2–21.3)	**6.7** * **(-0.21–13.5)**	28.5 (19.3–37.6)	N/A	N/A	N/A	N/A	N/A
Psychology only	N/A	N/A	10.6 (5.5–15.6)	33.1 (25.4–40.8)	12.3 (8.9–15.8)	**17.6** * **(11.3–23.9)**	36.6 (28.7–44.5)	N/A	N/A	N/A	N/A	N/A
**Country**												
England	65.0 (29.6–100.0)	**50.9** *** **(47.8–54.0)**	22.3 (1.4–43.3)	**32.5** *** **(16.8–48.3)**	**19.2** *** **(6.6–31.7)**	**7.7** *** **(-1.0–16.5)**	**31.8** *** **(17.7–45.8)**	40.4** **(29.0–51.8)**	**24.0** *** **(21.6–26.4)**	39.9 (13.1–66.7)	217.3 (11.3–43.4)	7.5* **(3.6–11.5)**
Northern Ireland	12.08 (11.7–70.5)	**12.1** *** **(-5.6–29.8)**	11.6 (5.8–17.4)	**18.1** *** **(14.5–21.7)**	**3.3** *** **(0.9–5.7)**	**2.9** *** **(1.7–4.1)**	**20.2** *** **(17.4–23.1)**	22.8** **(20.0–25.7)**	**9.3** *** **(2.1–16.4)**	31.1 (28.8–33.5)	16.2 (14.3–1821)	2.6* **(1.5–3.8)**
Scotland	N/A	N/A	10.6 (5.5–15.6)	**33.1** *** **(25.4–40.8)**	**11.3** *** **(6.1–16.5)**	**17.6** *** **(11.3–23.9)**	**36.6** *** **(28.7–44.5)**	N/A	N/A	N/A	N/A	N/A
Wales	N/A	N/A	N/A	N/A	**13.2** *** **(8.6–17.7)**	N/A	N/A	N/A	N/A	N/A	N/A	N/A
**Adversity measure**												
ACE scale	62.7** **(37.9–87.6)**	**41.1** *** **(18.5–63.7)**	18.2 (1.7–34.7)	28.9** **(14.8–42.9)**	**12.4** *** **(0.8–24.0)**	**8.2** *** **(- 0.04–16.5)**	28.5 (19.3–37.6)	N/A	17.3 (8.0–26.5)	12.4 (0.8–24.0)	23.5 (12.3–34.8)	N/A
Christoffersen et al., 2013	26.1** **(22.7–29.5)**	**3.1** *** **(1.8–4.5)**	10.6 (5.5–15.6)	33.1** **(25.4–40.8)**	**11.3** *** **(6.1–28.0)**	**17.6** *** **(11.3–23.9)**	36.6 (28.7–44.5)	N/A	N/A	N/A	N/A	N/A
CASE	N/A	N/A	N/A	N/A	**22.2** *** **(16.4–28.0)**	N/A	N/A	N/A	N/A	N/A	N/A	N/A
CTQ	N/A	N/A	16.9 (14.0–20.0)	19.2** **(16.2–22.3)**	**2.2** *** **(1.1–3.3)**	N/A	N/A	N/A	N/A	N/A	N/A	N/A
Oaksford & Frude, 2001	N/A	N/A	N/A	N/A	**13.2** *** **(8.6–17.7)**	N/A	N/A	N/A	N/A	N/A	N/A	N/A
TLEQ	N/A	N/A	11.8 (7.7–16.0)	20.6** **(15.4–25.9)**	N/A	**0.9** *** **(- 0.3–2.1)**	N/A	N/A	23.25 (17.8–28.7)	N/A	N/A	N/A
Army STARRS	N/A	N/A	N/A	N/A	N/A	N/A	N/A	N/A	N/A	32.2 (28.8–35.6)	15.7 (13.1–18.3)	N/A
# **Of adversities measured**												
Less than 10	**29.0** *** **(23.5–34.6)**	**3.1** *** **(1.8–4.5)**	N/A	N/A	N/A	N/A	N/A	N/A	N/A	N/A	N/A	N/A
10 or above	**73.0** *** **(56.0–90.0)**	**41.1** *** **(18.5–63.7)**	N/A	N/A	N/A	N/A	N/A	N/A	N/A	N/A	N/A	N/A
**Meta-regression–estimate (SE)**												
**Age**	0.03 (0.02)	0.02 (0.02)	**0.02** *** **(0.004)**	0.02** **(0.005)**	0.01* **(0.01)**	0.01 (0.01)	**0.02** *** **(0.003)**	**0.02** *** **(0.002)**	0.01** **(0.004)**	0.01 (0.006)	**0.01** *** **(0.003)**	**0.005** *** **(0.001)**
**% Of females**	**- 0.09** *** **(0.01)**	**- 0.08** *** **(0.01)**	0.001 (0.002)	0.001 (0.002)	0.001 (0.002)	0.001* **(0.002)**	0.004 (0.003)	N/A	- 0.001 (0.02)	-0.003 (0.005)	0.002 (0.003)	N/A

Note. Results in bold = significant results

* = significant at p < 0.05 level

** = significant at p < 0.01 level

*** = significant at p < 0.001 level; CI = confidence interval; SE = standard error; PA = physical abuse; EA = emotional abuse; SA = sexual abuse; PN = physical neglect; EN = emotional neglect; ACE = adverse childhood experience; CTQ = childhood trauma questionnaire; TLEQ = traumatic life events questionnaire; PS = parental separation; DV = domestic violence (between adults in childhood home); MHP = mental health problems (among adult(s) in childhood home); Sub = substance abuse (among adults(s) in childhood home); Inc = incarceration (lived with anyone who served time in prison during childhood); ACE scale = adverse childhood experiences scale; CASE = checklist to assess sexual exploitation; CTQ = childhood trauma questionnaire; TLEQ = traumatic life events questionnaire.

For the prevalence of three or more ACEs, a significant effect was found for the country the study was conducted in, with studies conducted in England (*n* = 2) reporting a higher prevalence of three or more ACEs than studies conducted in Northern Ireland (*n* = 2). Separately, the two studies conducted in England showed acceptable heterogeneity (I^2^ = 0.0%; p = 0.8) and a pooled prevalence of 51% (95% CI: 48.0–54.0%; Section A of S6 Appendix).

#### Childhood abuse

Random effects models suggested a weighted prevalence of 15.9% (95% CI: 7.0% - 24.7%), 27.0% (95% CI: 18.1% - 35.9%), and 12.1% (95% CI: 5.2% - 19.0%), and for PA, EA, and SA, respectively (Fig 2C–2E). Significant heterogeneity was observed between studies (I^2^ > 94.5%; p < .0001) for all meta-analyses. Leave-one-out analyses revealed no single study demonstrated an outsized effect on any of the pooled estimates (S4 Appendix). Quality effects models gave similar results, and even after removing Lagdon et al. [62] minimal differences were detected (Section B of S5 Appendix).

Tentative results are reported for country and measurement tool (Table 3) for SA and EA. Further subgroup analyses were conducted between the groups (S1 Table), in which for SA, they suggest that studies conducted in England (*n* = 4), Scotland (*n* = 1), and Wales (*n* = 1), reported significantly higher prevalence rates of SA compared to Northern Ireland (*n* = 3), and for EA, results show Scotland (*n* = 1) reports significantly higher prevalence rates of EA compared to Northern Ireland (*n* = 3).

In relation to the measurement tool, for SA, the CASE (*n* = 1) was associated with higher prevalence rates of SA compared to the CTQ (*n* = 1), questions developed by Christoffersen et al ([77]; *n* = 1), and questions developed by Oaksford and Frude ([66]; *n* = 1). Questions developed by Christoffersen et al. [77] and Oaksford and Frude [66] also showed significantly higher rates of SA compared to the CTQ. For EA, questions by Christoffersen et al. ([77]; *n* = 1) resulted in significantly higher rates of EA compared to the TLEQ (*n* = 1) and CTQ (*n* = 1). Given that the I^2^ value for subgroup analyses remained above 90%, and the low number of studies in each subgroup, results should be interpreted with caution.

#### Childhood neglect

Regarding the prevalence of childhood neglect (PN and EN), random effects models suggested a weighted prevalence of 8.4% (95% CI: 1.7% - 15.1%), and 30.0% (95% CI: 21.5% - 38.5%) for PN and EN, respectively (Fig 2(F) and 2(G). Significant heterogeneity was observed between studies (I^2^ > 95.4%; p < .0001) for both variables. Leave-one-out analyses revealed that no single study demonstrated an outsized effects on the pooled estimates (S4 Appendix). No appreciable differences were found when the quality effects models were run.

Regarding subgroup analyses, tentative significant effects were found for both PN and EN and the country the study was conducted in (Table 3). Further subgroup analyses (S1 Table) revealed that Scotland (*n* = 1) reported significantly higher PN and EN compared to Northern Ireland (*n* = 1). For PN, significant results are also reported for the type of students used in the research and the measurement tool used, whereby a higher prevalence of PN was found among a sample of Psychology students (*n* = 1) compared to a more general sample of university students from a range of disciplines (*n* = 5). Questions by Christoffersen et al. ([77]; *n* = 1) resulted in significantly higher rates of PN compared to the TLEQ (*n* = 1). Additionally, there were positive associations between the percentage of female participants and PN, and the age of participants and EN.

#### Household dysfunction

Random effects models suggested a weighted prevalence of 34.4% (95% CI: 22.8% - 46.0%), 18.4% (95% CI: 10.1% - 26.8%), 35.2% (95% CI: 23.6% - 46.8%), 21.4% (95% CI: 12.9% - 29.9%), and 5.7% (95% CI: 2.3% - 9.1%) for PS, DV, MHP, Sub, and Inc, respectively (Fig 2H-2L). Significant heterogeneity was observed between studies (I^2^ > 88.8%; p < .0001) for all. No appreciable differences were found when quality effects models were run, and even after removing O’Neil et al. [65] minimal differences were detected (Section C of S5 Appendix).

Leave-one-out analyses for PS and DV revealed that no single study demonstrated an outsized effect on the pooled estimates (S4 Appendix). For MHP and Sub, leave-one-out analyses revealed that one study [26] was having an outsized effect on the pooled estimates (S4 Appendix). Leaving this study out resulted in a pooled prevalence of 29.5% (95% CI: 26.2% - 32.8%; Section A of S7 Appendix) and 17.3% (95% CI: 15.1% - 19.5%; Section B of S7 Appendix) for MHP and Sub, respectively. Acceptable heterogeneity was also observed between studies for MHP (I^2^ = 69.9%; p = .04) and Sub (I^2^ = 52.2%; p = .12). Furthermore, for Inc, a leave-one-out analysis revealed that one study [63] was having an outsized effect on the pooled estimate (S4 Appendix). Leaving this study out resulted in a pooled prevalence of 7.5% (95% CI: 3.6% - 11.5%; Section B of S7 Appendix), with acceptable heterogeneity between studies (I^2^ = 62.7%; p = .10).

Regarding subgroup analyses, tentative results are reported for the country the study was conducted in (Table 3) and the prevalence of PS, DV, and Inc, whereby England (*n* = 2; *n* = 3; *n* = 2, respectively) reported higher prevalence rates compared to Northern Ireland (*n* = 1; *n* = 2; *n* = 1, respectively). For DV, the three studies conducted in England showed acceptable heterogeneity (I^2^ = 0.0%; p = 0.5) and a pooled prevalence of 24.0% (95% CI: 22.0–26.0%; Section B of S6 Appendix), and for Inc, the two studies conducted in England showed acceptable heterogeneity (I^2^ = 63.0%; p = 0.1) and a pooled prevalence of 8.0% (95% CI: 4.0–11.0%; Section C of S6 Appendix). Positive associations were found between the age of participants and PS, DV, Sub, and Inc.

#### Publication bias

Publication bias is usually assessed via visual inspection of the funnel plot and statistical tests for asymmetry [56]. It is recommended that statistical tests are used when there are at least ten studies included in the meta-analysis, due to a lack of power when fewer studies are included [78]. Given that there were less than ten studies in each of the meta-analysis, the statistical tests for funnel plot asymmetry were not deemed applicable. Funnel plots for each meta-analysis all visually suggest a degree of asymmetry (S8 Appendix); however, results should be taken with caution as low power makes it difficult to distinguish chance from real asymmetry [56].

## Discussion

The current systematic review and meta-analysis examines the prevalence of adverse childhood experiences (ACEs) among university students in the UK. Ten studies met the inclusion criteria and were included in the meta-analyses. High prevalence of ACEs were found among university students in the UK; however, results indicate high levels of uncertainty due to the degree of unexplained variability in the estimates of prevalence of ACEs among this population. Thus, the results should be taken with caution and are discussed tentatively.

### Main findings

This review suggests there may be a high prevalence of ACEs among university students in the UK (with the–albeit highly heterogeneous–data suggesting a pooled prevalence of over half (55.4%) reporting one or more ACE), although, the central tendency estimates cannot currently be interpreted with confidence. The high levels of heterogeneity and disparity echo findings for university students in China [42] with the pooled prevalence estimated for university students–also based on highly heterogeneous data–is even greater, at 64.7%. Confident interpretation of any differences between these estimates is not possible, but one might tentatively postulate geographical and cultural differences, as well as differences in inclusion criteria (as only studies which used a validated measure of childhood adversity were included in the previous review), as potential sources of difference.

The prevalence of at least one ACE in a general population sample in the UK has been estimated at 44.5% [6]. Again, conclusions must be highly tentative, but there is little in the current analysis to support the contention that those who attend university may be less likely than the general population to report ACEs, due to protective factors [19, 79]. These results provide support for the argument that university students should be given distinct research attention regarding the prevalence of ACEs [42], and further studies are required to explore this, as there appears to be no consensus within the literature at present.

This review also suggests high prevalence rates of childhood abuse and neglect among university students in the UK. The heterogeneous data suggests a pooled prevalence of 30.0%, 27.0%, 15.9%, 12.1%, and 8.4% for emotional neglect, emotional abuse, physical abuse, sexual abuse, and physical neglect, respectively. In comparison to a general population sample in the UK (emotional abuse—23%, physical abuse—14%, sexual abuse—6%; [6]), the prevalence of childhood abuse is higher among university students in the UK. Although tentative, these results further corroborate that those who attend university may be no less likely than the general population to report ACEs.

Regarding the prevalence of household dysfunction, the results suggest a pooled prevalence (albeit with highly heterogenous data) of 34.4% and 18.4% for parental separation and domestic violence, respectively. Despite the majority of ACEs being highly heterogenous, following a leave-one-out analysis, three ACEs relating to household dysfunction (mental health problems, substance use, and incarceration [among adults living in family home]) demonstrated acceptable heterogeneity. The pooled prevalence for these analyses were 29.5%, 17.3%, and 7.5% for mental health problems, substance use, and incarceration, respectively.

For mental health problems and substance use (among adults living in family home) removing the article by Martin-Denham and Donaghue ([26]; which represented the highest prevalence) resulted in acceptable heterogeneity. Despite the study with the highest prevalence being removed, the pooled prevalence of mental health problems and substance use were still higher than what was found in a general population sample in England (11% for mental health problem, 11% for alcohol use, and 4% for drug use; [6]). These results tentatively suggest that university students are no less likely to experience these ACEs than the general population, and that a sizeable proportion of university students in the UK may have grown up in an environment with parents/carers who struggled with their own mental health difficulties and may have used substances as a coping mechanism to manage their distress. Thus, it may be important for university support services to be aware of this when designing support services for students.

The high prevalence of mental health problems and substance use in the Martin-Denham and Donaghue [26] paper may be reflective of the location that data were collected, as although the authors are not aware of which specific university students attended, if data was collected from the authors’ affiliated university (Sunderland University) or other local universities in the North East, the high prevalence may link to the North-West of England reportedly having the highest rate of child poverty in the UK [80].

Regarding incarceration, removing the outlying paper with the lowest prevalence [62] also resulted in acceptable heterogeneity. The remaining papers [25, 26] potentially collected data from locations which had high rates of child poverty in the UK (Sunderland [39.7%] and Newham [49.5%; borough with the highest rate of child poverty in London]; [80]), whereas, McGavock & Spratt [63] collected data from Northern Ireland in 2010, which at that time reported lower child poverty rates of 21.4% [81]. It is therefore possible that the location from which data is collected underlies some of the heterogeneity found between studies, resulting in it being difficult to find overall prevalence rates of ACEs among the whole of the UK due to such disparities found among different areas.

This was further explored via subgroup analyses by including country of location as a factor. Potential location-related differences, particularly between England and Northern Ireland, and Scotland and Northern Ireland, were found for several ACEs. However, please note that this often involved pooling the England-based papers discussed above [25, 26]; thus, the same limitations apply here regarding the disparity between different areas. Additionally, in some instances, the Northern Ireland and Scotland samples consisted of only one paper/university, so may not be truly representative of each country. The one study conducted in Scotland [64] included female psychology students only and is therefore unlikely to be fully representative. These results must, of course, be taken only as potential indications that location *may* be a factor for consideration in future research/analysis, since the potential for confounds in the context of such small numbers of studies is extremely high.

Subgroup analysis also revealed that for three or more ACEs, domestic violence, and incarceration, acceptable heterogeneity was demonstrated for studies conducted in England. The pooled prevalence was 51%, 24.0%, and 8.0% for three or more ACEs, domestic violence, and incarceration, which are higher than prevalence rates among a general population sample in England ([6]; 17%, 16%, and 3%, respectively). Tentatively again, these results provide little evidence that those who attend university in England are less likely than the general population to report multiple ACEs, domestic violence, and incarceration. However, the limitations discussed above regarding papers by Davies et al. [25] and Martin-Denham and Donaghue [26] should be held in mind here.

Interestingly, the results showed that females reported lower prevalence of one or more, and three or more ACEs, which contradicts previous research that demonstrates females tend to report higher levels of ACEs in comparisons to males [82]. Haahr-Pedersen et al. [83] report that females are more likely to report a range of ACEs and were more likely than males to report childhood adversity related to a dysfunctional home life, which may help to explain this anomaly finding within this study. Of the studies which were included in the analyses, two of the studies [62, 67] had the highest percentage of females present in the sample, and the lowest prevalence rates of ACEs. However, these papers only included ACEs relating to childhood maltreatment and neglect; they did not account for household dysfunction or any other ACE. Thus, the range of ACEs were limited, and this result may be confounded by the number of ACEs measured, the measurement tool used, as well as location differences.

### Strengths and limitations

To the best of our knowledge, this is the first meta-analysis to consider a wide range of ACEs (including household dysfunction) among university students in the UK, as well as internationally, as the meta-analysis by Fu et al. [42] predominantly focused on childhood abuse and neglect among university students in China. Thus, this review and meta-analysis provides an initial glance into the prevalence of ACEs among university students in the UK, and demonstrates the needs for further research.

However, it is important to highlight that this meta-analysis does not come without its limitations. As discussed throughout, there are high levels of uncertainty among the results due to the degree of unexplained variability in the estimates of prevalence of ACEs among university students in the UK. Thus, the central tendency estimates cannot be interpreted with confidence and must be taken with caution.

Another limitation is regarding all subgroup and meta-regression analyses, as the low number of studies should be borne in mind. One difficulty with subgroup analyses is low power [75], particularly when there are low numbers of studies in the subgroups, unequal numbers within subgroups, and high heterogeneity between studies [76], all of which are relevant to the current analyses. It has been suggested that 3–4 times the number of studies of ‘average’ meta-analyses are required to have sufficient power within subgroup analyses [76]; however, some of the subgroups within these analyses contained only one study, and therefore should be taken with caution. Not only do subgroup analyses with insufficient power risk inflating Type 2 errors, they also potentially increase the risk of Type 1 errors due to several subgroup analyses being run for multiple different moderators [76], which could potentially result in chance findings [84]. Where study numbers are low, it becomes impossible to de-confound potentially relevant factors which could contribute to heterogeneity, including the location of data collection, age and sex of participants, assessment tool used, and many more, including inevitable idiosyncrasies at specific-study level.

More broadly, another possibly important factor in the discrepancies within the literature and difficulties analysing the data is a lack of universal agreement on the standard definition of childhood adversity, and the multiple different types of ACEs that are explored. For example, the results highlight that emotional neglect and emotional abuse may be important factors for further exploration among university students in the UK; however, there is a lack of clarity in the literature regarding the definition and measurement of emotional neglect and emotional abuse, with some research grouping them together [85]. Sheldon and colleagues [41] emphasise that such inconsistencies make it difficult to meaningfully synthesise and compare datasets. It has been argued that these discrepancies in definition may, in part, be linked to the lack of systematic measurement of ACEs and childhood trauma (and vice versa), which may have implications for screening and assessing ACEs [86, 87].

The variety of different measurement tools used to assess ACEs, and in some instances the lack of validation and/or psychometric properties reported, are important factors when considering the studies included in this meta-analysis and the limitations of the analyses. For example, some measures ask only one question for a particular ACE, which is thought to lead to underestimates of prevalence [88]. Measures (such as the ACE scale) which use dichotomous responses and count the total number of ACEs ignore variability in responses, as well as the timing, duration, impact, severity, and the meaning these experiences have for individuals [89, 90], in which concerns have been raised regarding the ACE score being misused as a screening or diagnostic tool [91].

The breadth of possible ACEs was limited in these meta-analyses due to inadequate data available for some areas, such as bullying and deprivation. Furthermore, the majority of measurement tools included did not take into account other experiences of adversity, such as discrimination, hate crime, racism, poverty etc. The self-report and retrospective nature of ACE measurement tools is another criticism of these tools, as people may underestimate the significance of the event, have memory biases, fail to correctly recall memories, or choose not to share such private information [92–94]. There was some evidence in the current analyses that the measurement tool used may affect reported prevalence, with the ACE scale reporting higher prevalence rates of overall ACEs, and multiple discrepancies between questions developed by authors and some of the more validated scales (such as the CTQ and TLEQ) for the prevalence of individual ACEs. However, again, with so few studies, it is impossible to de-confound factors relating to this variable from others of potential importance (e.g. location).

Another important limitation of this systematic review and meta-analysis (and of the studies included in the meta-analyses) is the conceptualisation of the transition to university, and how this is underpinned by potentially outdated views of university students transitioning to university and moving away from home. Although this may be the case for some students, it may not reflect the experience of around a quarter of students who are thought to live at home and commute to university [95], with the concept of hybrid/blended learning becoming more prevalent since COVID-19; [96]). Students from minoritised-racial groups, lower social class groups, and deprived areas are more likely to commute to university and have poorer outcomes than their counterparts [95]. Thus, when exploring the prevalence of ACEs among this population, it seems imperative to understand some of the wider systemic and societal factors surrounding students, and whether they stay at or commute to university.

### Implications for practice, policy, and research

Despite the aforementioned limitations, the findings have important implications for practice, policy, and research. One of the main difficulties when trying to synthesise ACE data is the discrepancies in definitions of childhood adversity and individual ACEs; therefore, a more unified and universally agreed definition would be beneficial for future research, as well as more cohesive and validated measurement tools to allow for better insights into the prevalence and impact of ACEs.

Whilst high levels of heterogeneity preclude confident interpretation of single central summary estimates, there were indications of high prevalence of ACEs within this current meta-analysis, implying that these are common experiences among university students in the UK. There was little evidence for the assumption that ACEs may be lower among this population, thus, consideration should be given by universities, policymakers, and researchers to further understand the prevalence and impact of ACEs among this population, and to offer support as early as possible to help minimise the detrimental impact of ACEs, support their mental well-being, and support academic studies [15, 16]. It may be useful for support services in universities to explore ACEs during assessment and formulation sessions with students, to help gain further understanding of some of their early life experiences, and to help conceptualise their current distress as useful and adaptive survival strategies as a result of their earlier experiences [97].

Additionally, there were tentative suggestions in the data of high prevalence of childhood emotional abuse and neglect among university students in the UK; however, emotional neglect is a largely under-represented area in the scientific research [98]. Therefore, given the detrimental impact that emotional neglect can have (including loneliness, a failure to thrive, low mood, low self-esteem, substance use, suicidal ideation; [99, 100]), it may be important for future research to explore the prevalence and impact of this among UK university students, and to consider these areas when designing and providing support services.

### Conclusion

In conclusion, the results suggest high prevalence rates of ACEs among university students in the UK, with little evidence in the current analyses supporting the contention that those who attend university may be less likely than the general population to report ACEs due to protective factors. However, this systematic review and meta-analysis demonstrates difficulties in provision of any “true” prevalence estimates of ACEs due to currently unexplained variability in estimates. Potential sources of heterogeneity, including measurement tools and location, should be considered in future work. Clearer universal definitions of childhood adversity and unified measurement tools may allow for better assessment, understanding, and synthesis of the prevalence and impact of ACEs among university students in the UK. These findings should spur future research into the prevalence and impact of ACEs among this population and for universities and policymakers to consider how best to support students with lived experience of ACEs to help minimise any detrimental impact on their mental well-being and academic studies.

## Supporting information

S1 Appendix Search strategy. (PDF)

S2 Appendix Risk of bias appraisal tool. (PDF)

S3 Appendix Risk of bias appraisal score. (PDF)

S4 Appendix Leave-one-out analyses. (PDF)

S5 Appendix Additional analyses. (PDF)

S6 Appendix Forest plots from subgroup analyses. (PDF)

S7 Appendix Forest plots from leave-one-out analyses. (PDF)

S8 Appendix Funnel plots. (PDF)

S9 Appendix Prospero protocol. (PDF)

S10 Appendix PRISMA checklist. (DOCX)

S1 Table Additional sub-group analyses. (PDF)

S1 Data Meta-analysis data set. (XLSX)
