# Supplementary material for: Exploring the prevalence of childhood adversity among university students in the United Kingdom: A systematic review and meta-analysis
Source: PLoS One. 2024 Aug 28;19(8):e0308038. doi: 10.1371/journal.pone.0308038 (PMC11356454; doi:10.1371/journal.pone.0308038)
Supplement: S1 Appendix — (PDF) [file pone.0308038.s001.pdf]

Search terms			Number of Results from Database<br><br>Filters applied:<br>- peer-reviewed journal<br>- English language<br>- Published between 2000-2024					
Primary search term (title, abstract, key words)	AND (title, abstract, key words)	AND (title, abstract, key words OR author affiliation/ location identifier)	**Psyc INFO**	**Pubmed**	**ERIC**	**BEI**	**AMED**	**CINAHL**
trauma*<br>OR<br>abus*<br>OR<br>maltreat*<br>OR<br>advers*<br>OR<br>neglect*<br>OR<br>ptsd<br>OR<br>post?traumatic stress<br>OR<br>distress*<br>OR<br>ACE*	universit*<br>OR<br>student*<br>OR<br>graduate*<br>OR<br>undergrad*<br>OR<br>postgrad*	united kingdom<br>OR<br>uk<br>OR<br>Britain<br>OR<br>british<br>OR<br>england<br>OR<br>scotland<br>OR<br>wales<br>OR<br>northern Ireland<br>OR<br>English<br>OR<br>Scottish<br>OR<br>Welsh<br>OR<br>Northern Irish	2042	1985	1158	181	22	988
Checked bibliography of related reviews and found relevant papers							2	
						**Total**	**6378**	
