# Supplementary material for: Exploring the prevalence of childhood adversity among university students in the United Kingdom: A systematic review and meta-analysis
Source: PLoS One. 2024 Aug 28;19(8):e0308038. doi: 10.1371/journal.pone.0308038 (PMC11356454; doi:10.1371/journal.pone.0308038)
Supplement: S2 Appendix — (PDF) [file pone.0308038.s002.pdf]

Table A. Risk of bias tool

Criteria	Quality Rating<br>0<br>Unclear<br>(red)	0<br>Poor<br>(yellow)	1<br>Adequate<br>(amber)	2<br>Good<br>(green)
**Sample**	Sample is described but details are unclear	Sample not specified<br><br>**AND/OR**<br><br>Mean age **and** sex of sample not specified	Single restricted or non-random sample e.g., psychology students only<br><br>**AND/OR**<br><br>Single regional sample e.g., one UK university<br><br>**AND**<br><br>Mean age **AND/OR** sex specified	Multiple restricted or non-random samples e.g., students from a range of disciplines /overall university sample<br><br>**AND/OR**<br><br>Multiple regions non-random sampling e.g., more than one UK university<br><br>**AND**<br><br>Mean age **AND** sex specified
**Clarity/consistency of definition of adverse experience with current definitions (according to assessment tool used)**	Lack of information available/unclear regarding how the assessment tool defines the adverse experience	The definition provided by the assessment tool has **large** discrepancies (e.g. markedly different age range specified) between the tools definition and the definition accepted in the current review (see below)	The definition provided by the assessment tool has **some** differences compared to the definition accepted in the current review, but largely aligns with the definition (see below)	Definition aligns well with the definition accepted in the current review (see below)
**Quality of adversity assessment tool**	Lack of information available/unclear regarding the assessment tool used and its psychometric properties	No psychometric properties of assessment tool reported	Psychometric properties of assessment tool reported in	Psychometric properties of assessment tool reported in

	**AND/OR**<br><br>A standardised assessment tool has not been used<br><br>**AND/OR**<br><br>Assessment tool has not previously been validated against another measure which assesses the specified adverse experience (e.g. sexual abuse) **OR** has found not to be valid/reliable.	current paper and are acceptable<br><br>**AND/OR**<br><br>Assessment tool has previously been validated against another measure which assesses the specified adverse experience (e.g. sexual abuse) **AND** has been found to be acceptably valid and reliable.	current paper and are acceptable<br><br>**AND**<br><br>Assessment tool has previously been validated against a ‘Gold Standard’ measure or ‘Clinical Interview’ which assesses the specified adverse experience (e.g. sexual abuse) **AND** has been found to be acceptably valid and reliable.

Table B. Accepted Definitions of Adverse Childhood Experiences

Type of adversity	Accepted definition
Childhood adversity	**Childhood adversity** refers to a wide range of circumstances or events which may pose a serious threat to a child's physical or psychological well-being. Examples include child abuse and neglect, household dysfunction, bullying, serious accidents, discrimination, extreme poverty, and community violence (Bartless & Sacks, 2019) usually occurring within the first 18 years of life.
**Childhood Abuse (within first 18 years of life)**	
Sexual abuse	Child sexual abuse is when a child is forced or enticed to take part in sexual activities. These activities may involve physical contact or non-contact activities, and they can happen online or offline (Department for Education [DFE], 2018).
Physical abuse	Physical abuse is any way of deliberately hurting a child which causes them physical harm (NSPCC, 2020).
Emotional abuse	Emotional abuse involves the ongoing emotional maltreatment of a child (DFE, 2018). It may sometimes be called psychological abuse.
**Neglect (within first 18 years of life)**	
Physical neglect	Involves a parent or carer failing to take care of the child's basic physical needs, including providing shelter, food, and clothing (DFE, 2018).
Emotional neglect	Involves a parent or carer failing to take care of the child's basic psychological/emotional needs, and protecting them from emotional harm (DFE, 2018).
**Household dysfunction (within first 18 years of life)**	
Parental separation	Parents divorcing or separating during childhood, regardless of the reason for separation (Petersen et al., 2016).
Domestic violence	Witnessing domestic violence (slap, hit, kick, punch, or beat each other up) from parents/carers during childhood (Felitti et al., 1998).
Mental health problem	Lived with anyone who experienced mental health difficulties and/or suicidal thoughts or behaviours during childhood (Felitti et al., 1998).
Substance abuse	Involves living in a household where adults were experiencing alcohol and/or drug use problems during childhood (Felitti et al., 1998).
Incarceration	Lived with anyone who served time in prison (or was sentenced to serve time in prison) during childhood (Felitti et al., 1998).
