# Supplementary material for: Exploring the prevalence of childhood adversity among university students in the United Kingdom: A systematic review and meta-analysis
Source: PLoS One. 2024 Aug 28;19(8):e0308038. doi: 10.1371/journal.pone.0308038 (PMC11356454; doi:10.1371/journal.pone.0308038)
Supplement: S3 Appendix — (PDF) [file pone.0308038.s003.pdf]

Author, Year of publication	Risk of bias criteria											
	1 or more adverse experience				3 or more adverse experiences				Sexual abuse			
	Sample	Definition	Tool	Score	Sample	Definition	Tool	Score	Sample	Definition	Tool	Score
Davies, Read & Shevlin, 2022				0.67				0.67				0.50
Gracie et al., 2007	-	-	-	-	-	-	-	-	-	-	-	-
Ireland, Alderson & Ireland, 2015	-	-	-	-	-	-	-	-				0.5
Lagdon et al., 2021*				0.17				0.17				0.17
Martin-Denham & Donaghue, 2022				0.67				0.67				0.50
McGavock & Spratt, 2017				0.67				0.67				0.50
Moulton et al., 2015	-	-	-	-	-	-	-	-				0.50
O'Neil et al., 2018	-	-	-	-	-	-	-	-				0.50
Oaksford & Frude, 2001	-	-	-	-	-	-	-	-				0.33
Worsley et al., 2018				0.67	-	-	-	-				0.50
	Physical abuse				Emotional abuse				Physical neglect			
**Author, Year of publication**	Sample	Definition	Tool	Score	Sample	Definition	Tool	Score	Sample	Definition	Tool	Score
Davies, Read & Shevlin, 2022	-	-	-	-				0.67				0.67
Gracie et al., 2007				0.67				0.67				0.67
Ireland, Alderson & Ireland, 2015	-	-	-	-	-	-	-	-	-	-	-	-
Lagdon et al., 2021				0.17				0.17	-	-	-	-
Martin-Denham & Donaghue, 2022				0.67				0.67				0.67
McGavock & Spratt, 2017				0.67				0.67				0.67
Moulton et al., 2015				0.67				0.67				0.67
O'Neil et al., 2018				0.67				0.67	-	-	-	-
Oaksford & Frude, 2001	-	-	-	-	-	-	-	-	-	-	-	-
Worsley et al., 2018				0.67				0.67				0.67

Note. Key for criteria scoring; Red = unclear (0); Yellow = poor (0); Amber = adequate (1); Green = good (2). * Looked at adversity under 12 years old only.

Author, Year of publication	Risk of bias criteria											
	Emotional neglect				Parental separation				Domestic violence			
	Sample	Definition	Tool	Score	Sample	Definition	Tool	Score	Sample	Definition	Tool	Score
Davies, Read & Shevlin, 2022				0.67				0.67				0.67
Gracie et al., 2007	-	-	-	-	-	-	-	-				0.67
Ireland, Alderson & Ireland, 2015	-	-	-	-	-	-	-	-	-	-	-	-
Lagdon et al., 2021	-	-	-	-	-	-	-	-	-	-	-	-
Martin-Denham & Donaghue, 2022				0.67				0.67				0.67
McGavock & Spratt, 2017				0.67				0.67				0.67
Moulton et al., 2015				0.67	-	-	-	-	-	-	-	-
O'Neil et al., 2018	-	-	-	-	-	-	-	-				0.67
Oaksford & Frude, 2001	-	-	-	-	-	-	-	-	-	-	-	-
Worsley et al., 2018				0.67	-	-	-	-	-	-	-	-

Author, Year of publication	Mental health problem				Substance use				Incarceration			
	Sample	Definition	Tool	Score	Sample	Definition	Tool	Score	Sample	Definition	Tool	Score
Davies, Read & Shevlin, 2022				0.67				0.67				0.67
Gracie et al., 2007	-	-	-	-	-	-	-	-	-	-	-	-
Ireland, Alderson & Ireland, 2015	-	-	-	-	-	-	-	-	-	-	-	-
Lagdon et al., 2021	-	-	-	-	-	-	-	-	-	-	-	-
Martin-Denham & Donaghue, 2022				0.67				0.67				0.67
McGavock & Spratt, 2017				0.67				0.67				0.67
Moulton et al., 2015	-	-	-	-	-	-	-	-	-	-	-	-
O'Neil et al., 2018				0.17				0.17	-	-	-	-
Oaksford & Frude, 2001	-	-	-	-	-	-	-	-	-	-	-	-
Worsley et al., 2018	-	-	-	-	-	-	-	-	-	-	-	-

Note. Key for criteria scoring; Red = unclear (0); Yellow = poor (0); Amber = adequate (1); Green = good (2).
