# Supplementary material for: Exploring the prevalence of childhood adversity among university students in the United Kingdom: A systematic review and meta-analysis
Source: PLoS One. 2024 Aug 28;19(8):e0308038. doi: 10.1371/journal.pone.0308038 (PMC11356454; doi:10.1371/journal.pone.0308038)
Supplement: S4 Appendix — (PDF) [file pone.0308038.s004.pdf]

One or More ACE

	PR	95%-CI	p-value	tau^2	tau	I^2
Omitting Davies, Read & Shevlin	0.4937	[0.2839; 0.7034]	< 0.0001	0.0454	0.2130	99.2%
Omitting Lagdon et al.	0.6272	[0.3785; 0.8759]	< 0.0001	0.0640	0.2530	99.5%
Omitting Martin-Denham & Donaghue	0.4831	[0.2302; 0.7360]	0.0002	0.0663	0.2575	99.6%
Omitting McGavock & Spratt	0.5523	[0.2565; 0.8481]	0.0003	0.0907	0.3012	99.7%
Omitting Worsley, McIntyre, Bentall & Corcoran	0.6131	[0.3497; 0.8765]	< 0.0001	0.0718	0.2680	99.5%

Three or More ACEs

	PR	95%-CI	p-value	tau^2	tau	I^2
Omitting Davies, Read & Shevlin	0.2496	[0.0553; 0.4438]	0.0118	0.0289	0.1699	99.2%
Omitting Lagdon et al.	0.4114	[0.1854; 0.6373]	0.0004	0.0392	0.1979	98.9%
Omitting Martin-Denham & Donaghue	0.2497	[-0.0195; 0.5189]	0.0691	0.0564	0.2375	99.7%
Omitting McGavock & Spratt	0.3516	[-0.0265; 0.7297]	0.0684	0.1110	0.3332	99.7%

Physical Abuse

	PR	95%-CI	p-value	tau^2	tau	I^2
Omitting Gracie et al.	0.1658	[0.0606; 0.2710]	0.0020	0.0168	0.1297	95.4%
Omitting Lagdon et al.	0.1575	[0.0510; 0.2639]	0.0037	0.0172	0.1311	94.6%
Omitting Martin-Denham & Donaghue	0.1145	[0.0863; 0.1428]	< 0.0001	0.0010	0.0316	87.1%
Omitting Moulton et al.	0.1677	[0.0636; 0.2719]	0.0016	0.0165	0.1285	95.4%
Omitting Worsley et al.	0.1663	[0.0609; 0.2716]	0.0020	0.0168	0.1297	95.4%
Omitting O'Neil et al.	0.1746	[0.0747; 0.2744]	0.0006	0.0151	0.1227	93.1%
Omitting McGavock & Spratt	0.1664	[0.0612; 0.2717]	0.0019	0.0168	0.1296	95.4%

Emotional Abuse

	PR	95%-CI	p-value	tau^2	tau	I^2
Omitting Davies, Read & Shevlin	0.2611	[0.1600; 0.3621]	< 0.0001	0.0180	0.1341	94.6%
Omitting Gracie et al.	0.2795	[0.1783; 0.3808]	< 0.0001	0.0181	0.1345	96.4%
Omitting Lagdon et al.	0.2818	[0.1814; 0.3822]	< 0.0001	0.0177	0.1331	96.3%
Omitting Martin-Denham & Donaghue	0.2295	[0.1776; 0.2813]	< 0.0001	0.0045	0.0668	93.6%
Omitting Moulton et al.	0.2622	[0.1616; 0.3629]	< 0.0001	0.0180	0.1342	96.2%
Omitting Worsley et al.	0.2789	[0.1769; 0.3809]	< 0.0001	0.0183	0.1352	96.4%
Omitting O'Neil et al.	0.2883	[0.1933; 0.3832]	< 0.0001	0.0158	0.1256	95.2%
Omitting McGavock & Spratt	0.2799	[0.1784; 0.3814]	< 0.0001	0.0181	0.1346	96.4%

Sexual Abuse

	PR	95%-CI	p-value	tau^2	tau	I^2
Omitting Davies, Read & Shevlin	0.1130	[0.0371; 0.1890]	0.0035	0.0116	0.1076	95.4%
Omitting Ireland, Alderson & Ireland	0.1093	[0.0366; 0.1820]	0.0032	0.0107	0.1032	97.1%
Omitting Lagdon et al.,	0.1343	[0.0609; 0.2077]	0.0003	0.0108	0.1038	97.4%
Omitting Martin-Denham & Donaghue	0.0953	[0.0415; 0.1491]	0.0005	0.0057	0.0757	96.8%
Omitting Moulton et al.	0.1229	[0.0447; 0.2012]	0.0021	0.0124	0.1113	97.4%
Omitting Oaksford & Frude	0.1206	[0.0423; 0.1989]	0.0025	0.0124	0.1113	97.4%
Omitting Worsley, McIntyre, Bentall & Corcoran	0.1334	[0.0592; 0.2077]	0.0004	0.0110	0.1050	97.4%
Omitting O'Neil et al\n	0.1345	[0.0612; 0.2077]	0.0003	0.0107	0.1035	97.3%
Omitting McGavock & Spratt	0.1297	[0.0529; 0.2066]	0.0009	0.0118	0.1088	97.5%

Physical Neglect

	PR	95%-CI	p-value	tau^2	tau	I^2
Omitting Davies, Read & Shevlin	0.0837	[0.0006; 0.1669]	0.0484	0.0086	0.0927	93.8%
Omitting Gracie et al.	0.0995	[0.0249; 0.1740]	0.0090	0.0068	0.0826	96.0%
Omitting Martin-Denham & Donaghue	0.0589	[0.0032; 0.1146]	0.0383	0.0038	0.0619	94.8%
Omitting Moulton et al.	0.0665	[-0.0021; 0.1351]	0.0574	0.0059	0.0768	95.4%
Omitting Worsley et al.	0.0981	[0.0217; 0.1745]	0.0119	0.0072	0.0848	95.9%
Omitting McGavock & Spratt	0.0957	[0.0168; 0.1747]	0.0175	0.0077	0.0877	96.3%

Emotional Neglect

	PR	95%-CI	p-value	tau^2	tau	I^2
Omitting Davies, Read & Shevlin	0.3043	[0.1977; 0.4109]	< 0.0001	0.0110	0.1048	96.5%
Omitting Martin-Denham & Donaghue	0.2494	[0.1793; 0.3195]	< 0.0001	0.0046	0.0680	94.6%
Omitting Moulton et al.	0.2848	[0.1931; 0.3764]	< 0.0001	0.0082	0.0908	96.9%
Omitting Worsley et al.	0.3368	[0.2334; 0.4402]	< 0.0001	0.0103	0.1013	95.4%
Omitting McGavock & Spratt	0.3291	[0.2079; 0.4502]	< 0.0001	0.0144	0.1201	97.2%

Parental Separation

	PR	95%-CI	p-value	tau^2	tau	I^2
Omitting Davies, Read & Shevlin	0.3450	[0.1095; 0.5804]	0.0041	0.0280	0.1672	96.8%
Omitting Martin-Denham & Donaghue	0.2891	[0.1684; 0.4098]	< 0.0001	0.0073	0.0857	96.7%
Omitting McGavock & Spratt	0.4037	[0.2895; 0.5179]	< 0.0001	0.0059	0.0770	86.4%

Domestic Violence

	PR	95%-CI	p-value	tau^2	tau	I^2
Omitting Davies, Read & Shevlin	0.1694	[0.0877; 0.2511]	< 0.0001	0.0064	0.0800	96.2%
Omitting Gracie et al.	0.1725	[0.0800; 0.2650]	0.0003	0.0085	0.0922	97.8%
Omitting Martin-Denham & Donaghue	0.1620	[0.0731; 0.2509]	0.0004	0.0079	0.0891	97.8%
Omitting O'Neil et al.	0.1997	[0.0776; 0.3218]	0.0013	0.0149	0.1222	98.1%
Omitting McGavock & Spratt	0.2163	[0.1450; 0.2876]	< 0.0001	0.0047	0.0687	92.9%

Mental health problem

	PR	95%-CI	p-value	tau^2	tau	I^2
Omitting Davies, Read & Shevlin	0.3832	[0.2383; 0.5281]	< 0.0001	0.0157	0.1253	93.5%
Omitting Martin-Denham & Donaghue	0.2950	[0.2617; 0.3283]	< 0.0001	0.0006	0.0245	69.9%
Omitting O'Neil et al	0.3644	[0.1995; 0.5293]	< 0.0001	0.0206	0.1434	95.1%
Omitting McGavock & Spratt	0.3715	[0.2113; 0.5316]	< 0.0001	0.0193	0.1391	95.3%

Substance Use

	PR	95%-CI	p-value	tau^2	tau	I^2
Omitting Davies, Read & Shevlin	0.2232	[0.0990; 0.3473]	0.0004	0.0115	0.1070	92.0%
Omitting Martin-Denham & Donaghue	0.1729	[0.1508; 0.1949]	< 0.0001	0.0002	0.0141	52.2%
Omitting O'Neil et al	0.2353	[0.1229; 0.3477]	< 0.0001	0.0093	0.0964	91.0%
Omitting McGavock & Spratt	0.2321	[0.1152; 0.3490]	< 0.0001	0.0101	0.1005	92.1%

Incarceration

	PR	95%-CI	p-value	tau^2	tau	I^2
Omitting Davies, Read & Shevlin	0.0607	[-0.0137; 0.1352]	0.1099	0.0026	0.0510	89.3%
Omitting Martin-Denham & Donaghue	0.0429	[0.0092; 0.0766]	0.0125	0.0005	0.0233	91.6%
Omitting McGavock & Spratt	0.0753	[0.0361; 0.1146]	0.0002	0.0006	0.0235	62.7%
