# Supplementary material for: Exploring the prevalence of childhood adversity among university students in the United Kingdom: A systematic review and meta-analysis
Source: PLoS One. 2024 Aug 28;19(8):e0308038. doi: 10.1371/journal.pone.0308038 (PMC11356454; doi:10.1371/journal.pone.0308038)
Supplement: S5 Appendix — (PDF) [file pone.0308038.s005.pdf]

A. Overall Number of ACEs

The quality effects models gave a similar weighted prevalence of 60.5% (95% CI: 36.1% - 84.9%) and 38.2% (95% CI: 12.3% - 64.1%) for one or more, and three or more, adverse experiences respectively, and significant heterogeneity was again observed between studies ($I^2 > 99.5\%$; $p < .0001$). The analysis was repeated with Lagdon et al. (2021) excluded due to low quality/risk of bias score; however, minimal differences were detected.

For one or more ACE, the random effects models (after removing Lagdon et al., 2021) gave a similar weighted prevalence of 62.7% (95% CI: 37.9% - 87.6%), with significant heterogeneity observed between studies ($I^2 = 99.5\%$; $p < .0001$).

For three or more ACEs the random effects models (after removing Lagdon et al., 2021) gave a similar weighted prevalence of 41.1% (95% CI: 18.5% - 63.7%) with significant heterogeneity observed between studies ($I^2 = 98.9\%$; $p < .0001$).

B. Childhood Abuse

The quality effects models gave a similar weighted prevalence of 12.9% (95% CI: 5.8% - 20.1%), 15.7% (95% CI: 6.5% - 25.0%), and 27.8% (95% CI: 18.6% - 37.1%) for SA, PA, and EA, respectively, and significant heterogeneity was observed between studies ($I^2 > 98.1\%$; $p < .0001$). When Lagdon et al. (2021) was excluded from the meta-analyses due to low quality/risk of bias score, minimal differences were detected.

The random effects models (after removing Lagdon et al., 2021) gave a similar weighted prevalence of 13.4% (95% CI: 6.1% - 20.8%) for SA. Significant heterogeneity was observed between studies ($I^2 = 97.4\%$; $p < .0001$).

The random effects models (after removing Lagdon et al., 2021) gave a similar weighted prevalence of 15.8% (95% CI: 5.1% - 26.4%) for PA. Significant heterogeneity was observed between studies ($I^2 = 94.6\%$; $p < .0001$).

The random effects models (after removing Lagdon et al., 2021) gave a similar weighted prevalence of 28.2% (95% CI: 18.1% - 38.2%) for EA. Significant heterogeneity was observed between studies ($I^2 = 96.3\%$; $p < .0001$).

C. Household Dysfunction

The quality effects models gave a similar weighted prevalence of 35.9% (95% CI: 23.5% - 48.4%) and 22.8% (95% CI: 13.6% - 32.0%) for MI and Sub, respectively, whereby significant heterogeneity was observed between studies ($I^2 > 96.5\%$; $p < .0001$). No differences were found for PS, DV, and Inc when the quality effects models were run. In a further analysis, O'Neil et al. (2018) was excluded from MI and Sub meta-analyses due to low quality/risk of bias score; however, minimal differences were detected.

The random effects models (after removing O'Neil et al., 2018) gave a similar weighted prevalence of 36.4% (95% CI: 20.0% - 52.9%) for MI. Significant heterogeneity was observed between studies ($I^2 = 95.1\%$; $p < .0001$).

The random effects models (after removing O'Neil et al., 2018) gave a similar weighted prevalence of 23.5% (95% CI: 12.3% - 34.8%) for Sub. Significant heterogeneity was observed between studies ($I^2 = 91.0\%$; $p < .0001$).
