# Supplementary figures and images for: Exploring the prevalence of childhood adversity among university students in the United Kingdom: A systematic review and meta-analysis
Source: PLoS One. 2024 Aug 28;19(8):e0308038. doi: 10.1371/journal.pone.0308038 (PMC11356454; doi:10.1371/journal.pone.0308038)

## A. Three or More ACEs and Country

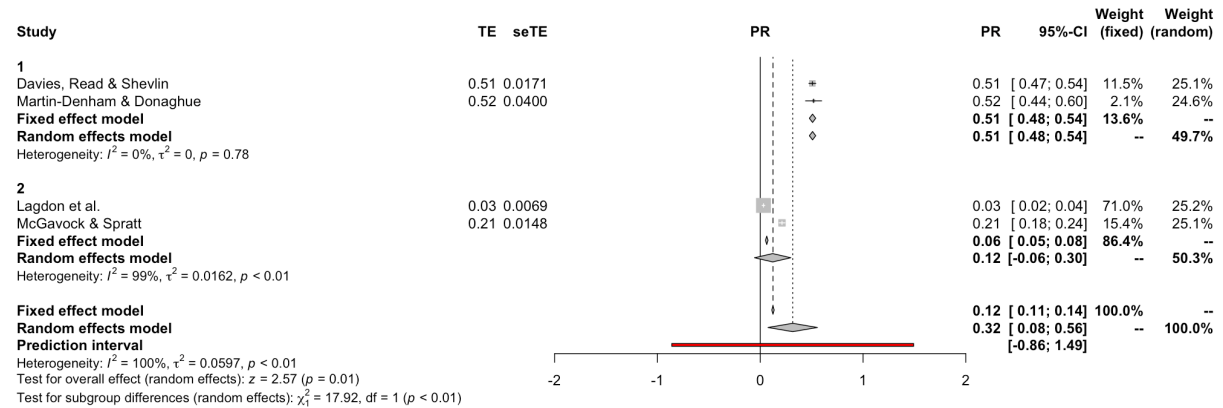

## B. Domestic Violence and Country

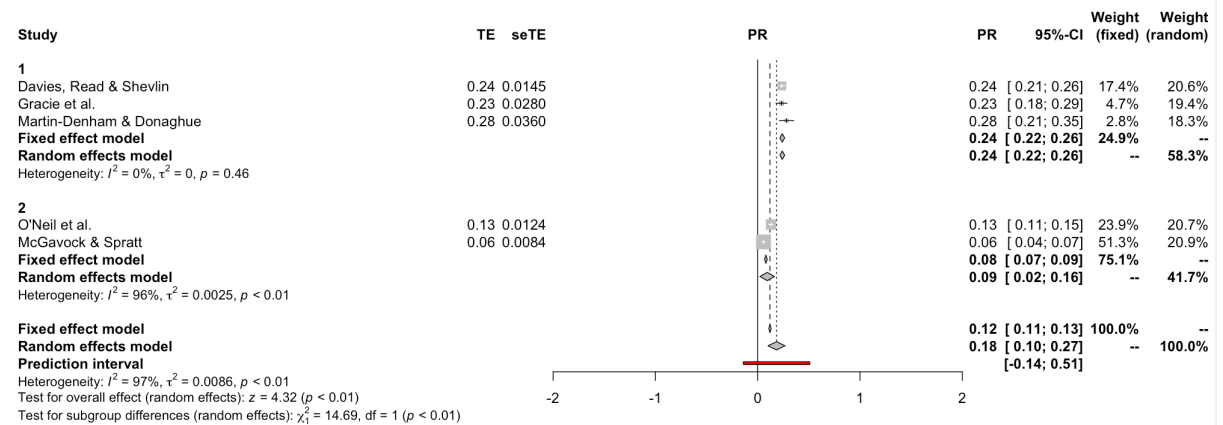

## C. Incarceration and Country

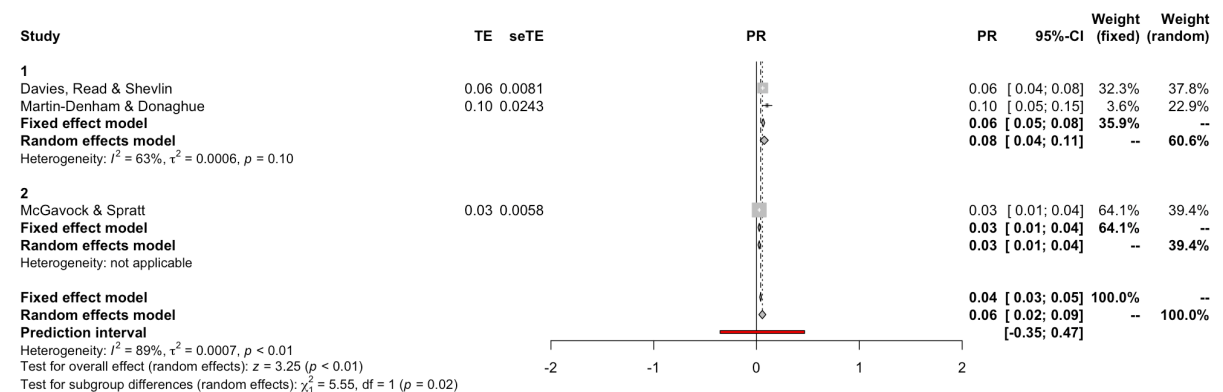

Supplement: S6 Appendix — (PDF) [file pone.0308038.s006.pdf]

## A. Mental Health Problems

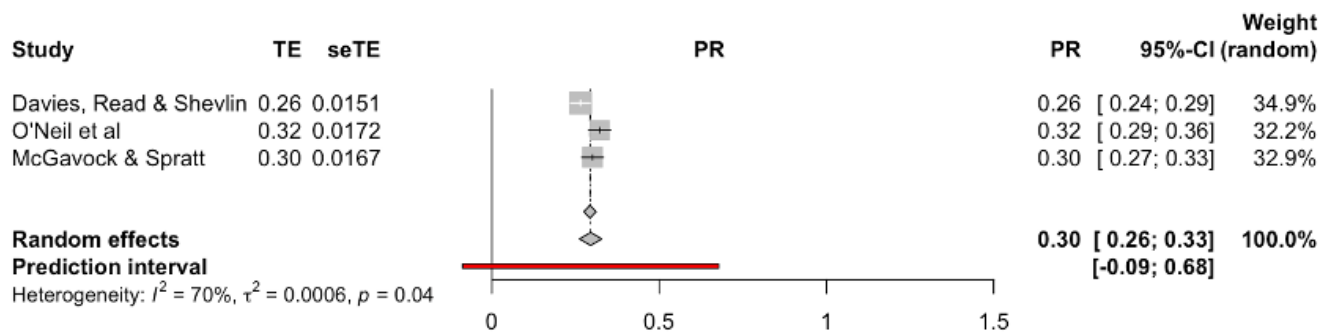

## B. Substance Use

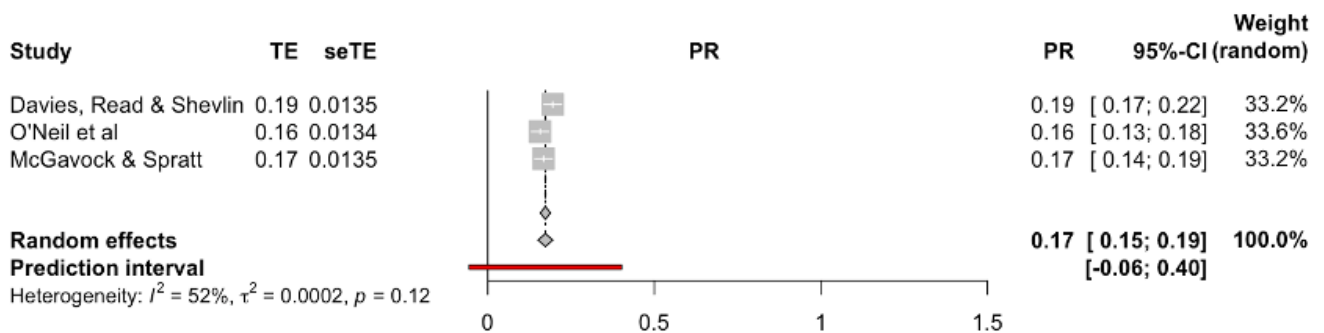

## C. Incarceration

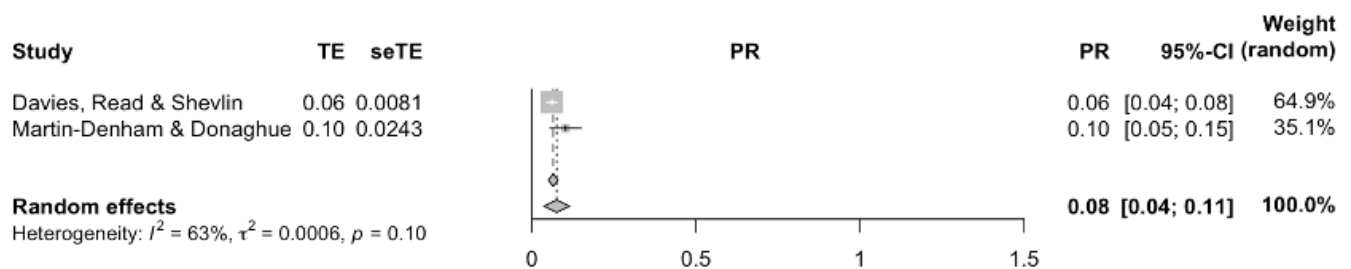

Supplement: S7 Appendix — (PDF) [file pone.0308038.s007.pdf]
