# Supplementary material for: Exploring the prevalence of childhood adversity among university students in the United Kingdom: A systematic review and meta-analysis
Source: PLoS One. 2024 Aug 28;19(8):e0308038. doi: 10.1371/journal.pone.0308038 (PMC11356454; doi:10.1371/journal.pone.0308038)

One or More ACE

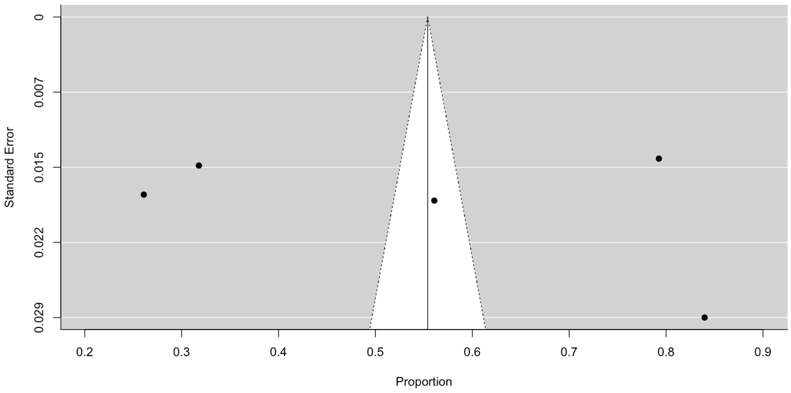

Three or More ACEs

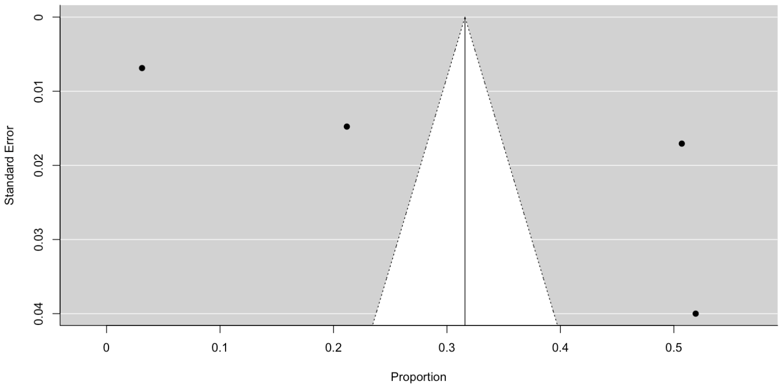

Sexual Abuse

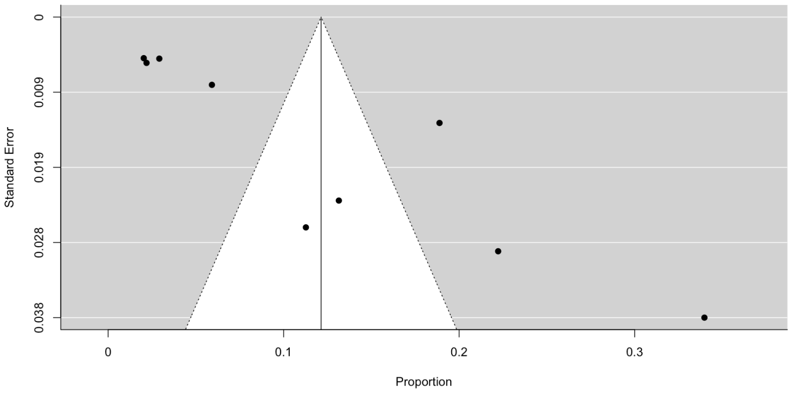

Physical Abuse

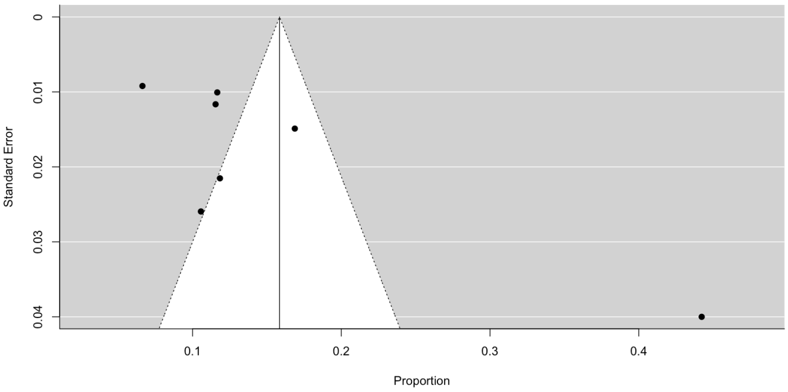

Emotional Abuse

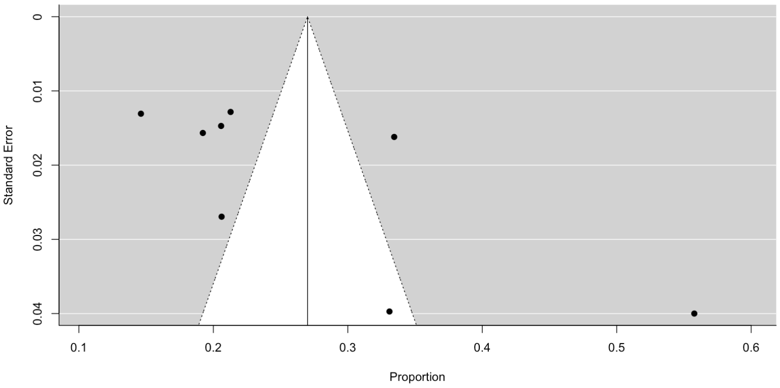

Physical Neglect

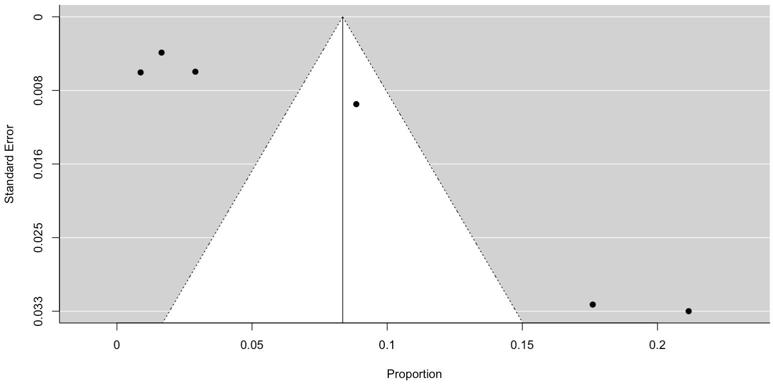

Emotional Neglect

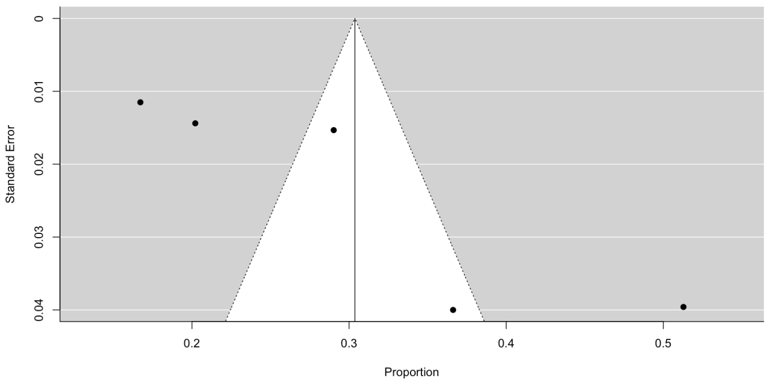

Parental Separation

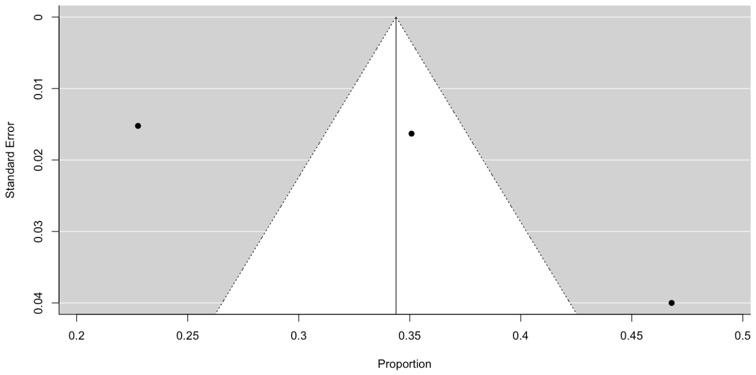

Domestic Violence

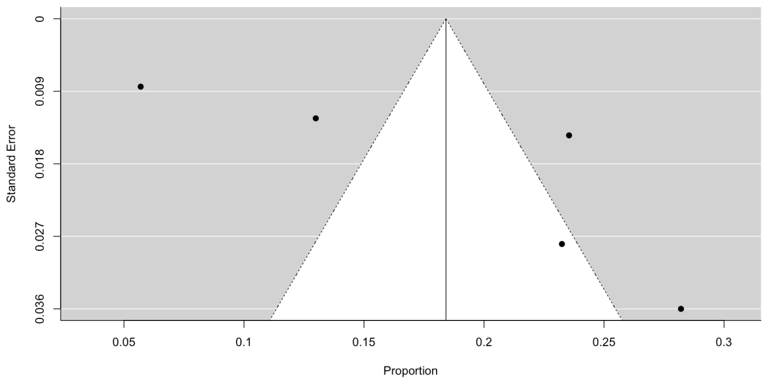

Mental health problem

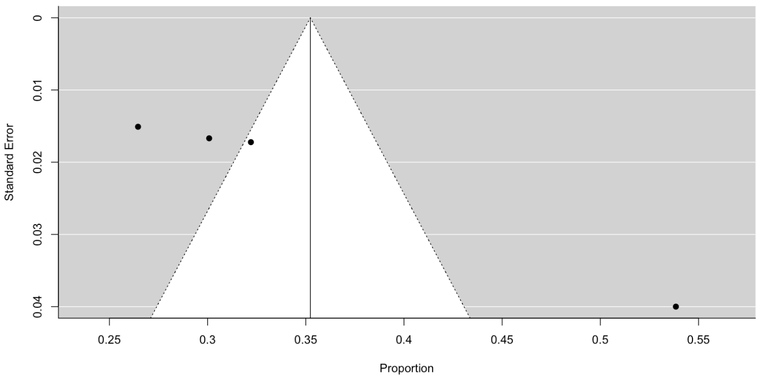

Substance Use

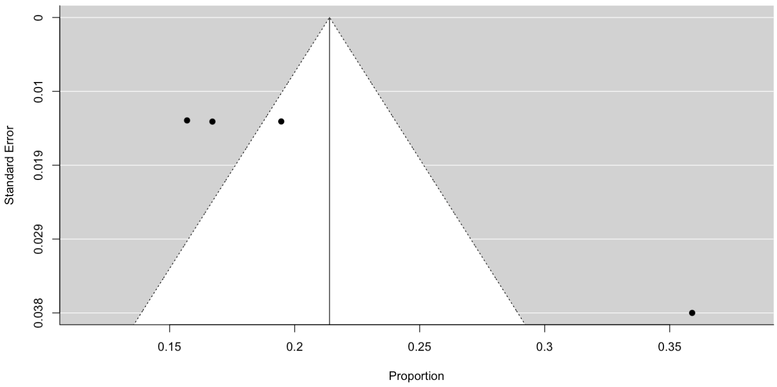

Incarceration

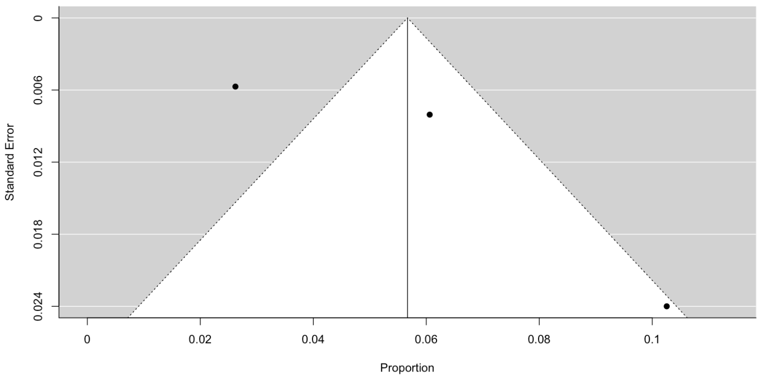

Supplement: S8 Appendix — (PDF) [file pone.0308038.s008.pdf]
