# Supplementary material for: Exploring the prevalence of childhood adversity among university students in the United Kingdom: A systematic review and meta-analysis
Source: PLoS One. 2024 Aug 28;19(8):e0308038. doi: 10.1371/journal.pone.0308038 (PMC11356454; doi:10.1371/journal.pone.0308038)
Supplement: S9 Appendix — (PDF) [file pone.0308038.s009.pdf]

National Institute for Health Research

UNIVERSITY of York
Centre for Reviews and Dissemination

Systematic review

A list of fields that can be edited in an update can be found here

1. * Review title.

Give the title of the review in English

Exploring the prevalence of childhood adversity among university students in the United Kingdom: A systematic review and meta-analysis

2. Original language title.

For reviews in languages other than English, give the title in the original language. This will be displayed with the English language title.

3. * Anticipated or actual start date.

Give the date the systematic review started or is expected to start.

19/08/2022

4. * Anticipated completion date. [1 change]

Give the date by which the review is expected to be completed.

31/03/2024

5. * Stage of review at time of this submission. [1 change]

This field uses answers to initial screening questions. It cannot be edited until after registration.

Tick the boxes to show which review tasks have been started and which have been completed.

Update this field each time any amendments are made to a published record.

The review has not yet started: No

Review stage	Started	Completed
Preliminary searches	Yes	Yes
Piloting of the study selection process	Yes	Yes
Formal screening of search results against eligibility criteria	Yes	Yes
Data extraction	Yes	Yes
Risk of bias (quality) assessment	Yes	Yes
Data analysis	Yes	Yes

Provide any other relevant information about the stage of the review here.

6. * Named contact.

The named contact is the guarantor for the accuracy of the information in the register record. This may be any member of the review team.

Jackie Hamilton

Email salutation (e.g. "Dr Smith" or "Joanne") for correspondence:

Dr Hamilton

7. * Named contact email.

Give the electronic email address of the named contact.

jackiehamilton26@googlemail.com

8. Named contact address

Give the full institutional/organisational postal address for the named contact.

DClinPsy Course, George Davies Centre, The University of Leicester, University Road, Leicester, LE1 7RH, United Kingdom

9. Named contact phone number.

Give the telephone number for the named contact, including international dialling code.

07581159440

10. * Organisational affiliation of the review.

Full title of the organisational affiliations for this review and website address if available. This field may be completed as 'None' if the review is not affiliated to any organisation.

University of Leicester

Organisation web address:

11. * Review team members and their organisational affiliations.

Give the personal details and the organisational affiliations of each member of the review team. Affiliation refers to groups or organisations to which review team members belong. **NOTE: email and country now MUST be entered for each person, unless you are amending a published record.**

Dr Jackie Hamilton. University of Leicester
Dr Alice Welham. University of Leicester

12. * Funding sources/sponsors.

Details of the individuals, organizations, groups, companies or other legal entities who have funded or sponsored the review.

Not applicable

Grant number(s)

State the funder, grant or award number and the date of award

13. * Conflicts of interest.

List actual or perceived conflicts of interest (financial or academic).

None

14. Collaborators.

Give the name and affiliation of any individuals or organisations who are working on the review but who are not listed as review team members. **NOTE: email and country must be completed for each person, unless you are amending a published record.**

15. * Review question.

State the review question(s) clearly and precisely. It may be appropriate to break very broad questions down into a series of related more specific questions. Questions may be framed or refined using PI(E)COS or similar where relevant.

What is known about the prevalence of childhood adversity among university students in the UK?

16. * Searches.

State the sources that will be searched (e.g. Medline). Give the search dates, and any restrictions (e.g. language or publication date). Do NOT enter the full search strategy (it may be provided as a link or

attachment below.)

The following six databases will be searched: Allied and Complementary Medicine (AMED); British Education Index (BEI); Cumulative Index to Nursing and Allied Health Literature (CINAHL); Education Resources Information Center (ERIC); MEDLINE; and PsycINFO.

Search dates: 2000 - current

Restrictions: English language only and peer-reviewed articles

17. URL to search strategy.

Upload a file with your search strategy, or an example of a search strategy for a specific database, (including the keywords) in pdf or word format. In doing so you are consenting to the file being made publicly accessible. Or provide a URL or link to the strategy. Do NOT provide links to your search **results**.

Alternatively, upload your search strategy to CRD in pdf format. Please note that by doing so you are consenting to the file being made publicly accessible.

Do not make this file publicly available until the review is complete

18. * Condition or domain being studied.

Give a short description of the disease, condition or healthcare domain being studied in your systematic review.

Adverse childhood experiences

19. * Participants/population.

Specify the participants or populations being studied in the review. The preferred format includes details of both inclusion and exclusion criteria.

University students from the United Kingdom

20. * Intervention(s), exposure(s).

Give full and clear descriptions or definitions of the interventions or the exposures to be reviewed. The preferred format includes details of both inclusion and exclusion criteria.

Exploring the prevalence of adverse childhood experiences among this population, and do a meta-analysis if possible.

Inclusion criteria (all criteria must be met for inclusion in review)

• University student sample

• Location: United Kingdom

• Assessment of exposure to childhood adversity (adverse experience before the age of 18 years old)

• Quantitative methodology

Exclusion criteria (paper excluded if any of the following criteria were met)

• Mixed sample of students and non-students, whereby results are not reported separately

• Case studies (due to limited generalisability)

• Assessment of lifetime exposure to adversity whereby childhood and adult adversity are reported as one

21. * Comparator(s)/control.

Where relevant, give details of the alternatives against which the intervention/exposure will be compared (e.g. another intervention or a non-exposed control group). The preferred format includes details of both inclusion and exclusion criteria.

Not applicable

22. * Types of study to be included.

Give details of the study designs (e.g. RCT) that are eligible for inclusion in the review. The preferred format includes both inclusion and exclusion criteria. If there are no restrictions on the types of study, this should be stated.

Inclusion criteria (all criteria must be met for inclusion in review)

• University student sample

• Location: United Kingdom

• Assessment of exposure to childhood adversity (adverse experience before the age of 18 years old)

• Quantitative methodology

Exclusion criteria (paper excluded if any of the following criteria were met)

• Mixed sample of students and non-students, whereby results are not reported separately

• Case studies (due to limited generalisability)

• Assessment of lifetime exposure to adversity whereby childhood and adult adversity are reported as one

23. Context.

Give summary details of the setting or other relevant characteristics, which help define the inclusion or exclusion criteria.

The aim of the review is to explore the prevalence of childhood adversity among university students in the UK. Quantitative data has been chosen in order to look at prevalence rates and conduct a meta-analysis. Inclusion criteria include university students and the UK as the location.

24. * Main outcome(s).

Give the pre-specified main (most important) outcomes of the review, including details of how the outcome is defined and measured and when these measurement are made, if these are part of the review inclusion criteria.

Main focus is on the prevalence of childhood adversity among the population.

Measures of effect

Please specify the effect measure(s) for you main outcome(s) e.g. relative risks, odds ratios, risk difference, and/or 'number needed to treat.

25. * Additional outcome(s).

List the pre-specified additional outcomes of the review, with a similar level of detail to that required for main outcomes. Where there are no additional outcomes please state 'None' or 'Not applicable' as appropriate to the review

To conduct a meta-analysis of prevalence rates if possible

Measures of effect

Please specify the effect measure(s) for you additional outcome(s) e.g. relative risks, odds ratios, risk difference, and/or 'number needed to treat.

26. * Data extraction (selection and coding).

Describe how studies will be selected for inclusion. State what data will be extracted or obtained. State how this will be done and recorded.

Studies will be selected according to the following inclusion/exclusion criteria:

Inclusion criteria (all criteria must be met for inclusion in review)

• University student sample

• Location: United Kingdom

• Assessment of exposure to childhood adversity (adverse experience before the age of 18 years old)

• Quantitative methodology

Exclusion criteria (paper excluded if any of the following criteria were met)

• Mixed sample of students and non-students, whereby results are not reported separately

• Case studies (due to limited generalisability)

• Assessment of lifetime exposure to adversity whereby childhood and adult adversity are reported as one

Data will be extracted into a table specifically designed for this review, and will include: title, authors, year, aims, participants information (n, age, gender, ethnicity), measures used, results (relating to prevalence of individual adversities and cumulative number).

27. * Risk of bias (quality) assessment.

State which characteristics of the studies will be assessed and/or any formal risk of bias/quality assessment tools that will be used.

Given that the main focus is regarding prevalence, an appraisal tool which was designed specifically for use in systematic reviews addressing questions of prevalence will be used - an amended version of Richards et al. (2015) criteria will be used, adapted for the current review.

Second author (Alice Welham) will independently score the papers too ad inter-rater reliability will be assessed.

28. * Strategy for data synthesis.

Describe the methods you plan to use to synthesise data. This **must not be generic text** but should be **specific to your review** and describe how the proposed approach will be applied to your data. If meta-analysis is planned, describe the models to be used, methods to explore statistical heterogeneity, and software package to be used.

The data will be synthesised narratively.

Dependant upon the data available, a meta-analysis will be conducted if possible. This will be on the prevalence data of adverse childhood experiences. It is proposed that a pooled prevalence meta-analysis will be conducted for each category of adverse experience (e.g., physical abuse; sexual abuse) in which two or more papers report prevalence data, using a random effects model to allow for between-study variation in possible effects (e.g., Borenstein, 2009; implemented by Jones, 2019, using the generic inverse variance method).

29. * Analysis of subgroups or subsets.

State any planned investigation of ‘subgroups’. Be clear and specific about which type of study or participant will be included in each group or covariate investigated. State the planned analytic approach.

Not applicable

30. * Type and method of review.

Select the type of review, review method and health area from the lists below.

Type of review

Cost effectiveness

No

Diagnostic

No

Epidemiologic

No

Individual patient data (IPD) meta-analysis

No

Intervention

No

Living systematic review

No

Meta-analysis

Yes

Methodology

No

Narrative synthesis

Yes

Network meta-analysis

No

Pre-clinical

No

Prevention

No

Prognostic

No

Prospective meta-analysis (PMA)

No

Review of reviews

No

Service delivery

No

Synthesis of qualitative studies

No

Systematic review
Yes

Other
No

Health area of the review

Alcohol/substance misuse/abuse
No

Blood and immune system
No

Cancer
No

Cardiovascular
No

Care of the elderly
No

Child health
No

Complementary therapies
No

COVID-19
No

Crime and justice
No

Dental
No

Digestive system
No

Ear, nose and throat
No

Education
No

Endocrine and metabolic disorders
No

Eye disorders

No

General interest

No

Genetics

No

Health inequalities/health equity

No

Infections and infestations

No

International development

No

Mental health and behavioural conditions

No

Musculoskeletal

No

Neurological

No

Nursing

No

Obstetrics and gynaecology

No

Oral health

No

Palliative care

No

Perioperative care

No

Physiotherapy

No

Pregnancy and childbirth

No

Public health (including social determinants of health)

Yes

Rehabilitation

No

Respiratory disorders

No

Service delivery

No

Skin disorders

No

Social care

No

Surgery

No

Tropical Medicine

No

Urological

No

Wounds, injuries and accidents

No

Violence and abuse

No

31. Language.

Select each language individually to add it to the list below, use the bin icon to remove any added in error.

English

There is not an English language summary

32. * Country.

Select the country in which the review is being carried out. For multi-national collaborations select all the countries involved.

England

33. Other registration details.

Name any other organisation where the systematic review title or protocol is registered (e.g. Campbell, or The Joanna Briggs Institute) together with any unique identification number assigned by them. If extracted data will be stored and made available through a repository such as the Systematic Review Data Repository (SRDR), details and a link should be included here. If none, leave blank.

34. Reference and/or URL for published protocol.

If the protocol for this review is published provide details (authors, title and journal details, preferably in Vancouver format)

Add web link to the published protocol.

Or, upload your published protocol here in pdf format. Note that the upload will be publicly accessible.

No I do not make this file publicly available until the review is complete

Please note that the information required in the PROSPERO registration form must be completed in full even if access to a protocol is given.

35. Dissemination plans.

Do you intend to publish the review on completion?

Yes

Give brief details of plans for communicating review findings.?

The review and meta-analysis will aim to be published in a peer review journal and in my DClinPsy thesis.

36. Keywords.

Give words or phrases that best describe the review. Separate keywords with a semicolon or new line. Keywords help PROSPERO users find your review (keywords do not appear in the public record but are included in searches). Be as specific and precise as possible. Avoid acronyms and abbreviations unless these are in wide use.

adversity; adverse childhood experiences; trauma; childhood trauma; university students; United Kingdom;

37. Details of any existing review of the same topic by the same authors.

If you are registering an update of an existing review give details of the earlier versions and include a full bibliographic reference, if available.

38. * Current review status. [1 change]

Update review status when the review is completed and when it is published.New registrations must be ongoing so this field is not editable for initial submission.

Please provide anticipated publication date

Review_Completed_not_published

39. Any additional information.

Provide any other information relevant to the registration of this review.

40. Details of final report/publication(s) or preprints if available.

Leave empty until publication details are available OR you have a link to a preprint (NOTE: this field is not editable for initial submission). List authors, title and journal details preferably in Vancouver format.

Give the link to the published review or preprint.
