# Supplementary material for: Exploring the prevalence of childhood adversity among university students in the United Kingdom: A systematic review and meta-analysis
Source: PLoS One. 2024 Aug 28;19(8):e0308038. doi: 10.1371/journal.pone.0308038 (PMC11356454; doi:10.1371/journal.pone.0308038)
Supplement: S1 Table — (PDF) [file pone.0308038.s011.pdf]

Study Characteristics	Prevalence % (95% CI)			
	SA	EA	PN	EN
**Country**				
**England vs Northern Ireland**				
England	**19.2* (6.6 – 31.7)**	32.5 (16.8 – 48.3)	7.7 (-1.0 – 16.5)	31.8 (17.7 – 45.8)
Northern Ireland	**3.3* (0.9 – 5.7)**	18.1 (14.5 – 21.7)	2.9 (1.7 – 4.1)	20.2 (17.4 – 23.1)
**England vs Scotland**				
England	19.2 (6.6 – 31.7)	32.5 (16.8 – 48.3)	7.7 (-1.0 – 16.5)	31.8 (17.7 – 45.8)
Scotland	11.3 (6.1 – 16.5)	33.1 (25.4 – 40.8)	17.6 (11.3 – 23.9)	36.6 (28.7 – 44.5)
**England vs Wales**				
England	19.2 (6.6 – 31.7)	N/A	N/A	
Wales	13.2 (8.6 – 17.7)	N/A	N/A	
**Northern Ireland vs Scotland**				
Northern Ireland	**3.3** (0.9 – 5.7)**	**18.1*** (14.5 – 21.7)**	**2.9*** (1.7 – 4.1)**	**20.2*** (17.4 – 23.1)**
Scotland	**11.3** (6.1 – 16.5)**	**33.1*** (25.4 – 40.8)**	**17.6*** (11.3 – 23.9)**	**36.6*** (28.7 – 44.5)**
**Northern Ireland vs Wales**				
Northern Ireland	**3.3*** (0.9 – 5.7)**	N/A	N/A	N/A
Wales	**13.2*** (8.6 – 17.7)**	N/A	N/A	N/A
**Scotland vs Wales**				
Scotland	11.3 (6.1 – 16.5)	N/A	N/A	N/A
Wales	13.2 (8.6 – 17.7)	N/A	N/A	N/A
**Measurement tool**				
**ACE vs Christoffersen et al., 2013**				
ACE	12.4 (0.8 – 24.0)	28.9 (14.8 – 42.9)	8.2 (-0.04 – 16.5)	N/A
Christoffersen et al., 2013	11.3 (6.1 – 28.0)	33.1 (25.4 – 40.8)	17.6 (11.3 – 23.9)	N/A
**ACE vs CASE**				
ACE	12.4 (0.8 – 24.0)	N/A	N/A	N/A
CASE	22.2 (16.4 – 28.0)	N/A	N/A	N/A
**ACE vs CTQ**				
ACE	12.4 (0.8 – 24.0)	28.9 (14.8 – 42.9)	N/A	N/A
CTQ	2.2 (1.1 – 3.3)	19.2 (16.2 – 22.3)	N/A	N/A
**ACE vs Oaksford & Frude, 2001**				
ACE	12.4 (0.8 – 24.0)	N/A	N/A	N/A
Oaksford & Frude, 2001	13.2 (8.6 – 17.7)	N/A	N/A	N/A
**ACE vs TLEQ**				
ACE		28.9 (14.8 – 42.9)	8.2 (-0.04 – 16.5)	N/A
TLEQ		20.6 (15.4 – 25.9)	0.9 (-0.3 – 2.1)	N/A
**Christoffersen et al., 2013 vs CASE**				
Christoffersen et al., 2013	**11.3 (6.1 – 28.0)****	N/A	N/A	N/A
CASE	**22.2 (16.4 – 28.0)****	N/A	N/A	N/A
**Christoffersen et al., 2013 vs CTQ**				
Christoffersen et al., 2013	**11.3 (6.1 – 28.0)*****	**33.1** (25.4 – 40.8)**	N/A	N/A
CTQ	**2.2 (1.1 – 3.3)*****	**19.2** (16.2 – 22.3)**	N/A	N/A
**Christoffersen et al., 2013 vs Oaksford & Frude, 2001**				
Christoffersen et al., 2013	11.3 (6.1 – 28.0)	N/A	N/A	N/A
Oaksford & Frude, 2001	13.2 (8.6 – 17.7)	N/A	N/A	N/A
**Christoffersen et al., 2013 vs TLEQ**				
Christoffersen et al., 2013		**33.1** (25.4 – 40.8)**	**17.6*** (11.3 – 23.9)**	N/A
TLEQ		**20.6** (15.4 – 25.9)**	**0.9*** (-0.3 – 2.1)**	N/A
**CASE vs CTQ**				
CASE	**22.2 (16.4 – 28.0)*****	N/A	N/A	N/A
CTQ	**2.2 (1.1 – 3.3)*****	N/A	N/A	N/A
**CASE vs Oaksford & Frude, 2001**				
CASE	**22.2 (16.4 – 28.0)***	N/A	N/A	N/A
Oaksford & Frude, 2001	**13.2 (8.6 – 17.7)***	N/A	N/A	N/A
**CTQ vs Oaksford & Frude, 2001**				
CTQ	**2.2 (1.1 – 3.3)*****	N/A	N/A	N/A
Oaksford & Frude, 2001	**13.2 (8.6 – 17.7)*****	N/A	N/A	N/A
**CTQ vs TLEQ**				
CTQ		19.2 (16.2 – 22.3)	N/A	N/A
TLEQ		20.6 (15.4 – 25.9)	N/A	N/A

Note. Results in bold = significant results; * = significant at p < 0.05 level; ** = significant at p < 0.01 level; *** = significant at p < 0.001 level; CI = confidence interval; SA = sexual abuse; EA; emotional abuse; PN = physical neglect; EN = emotional neglect; ACE scale = adverse childhood experiences scale; CASE = checklist to assess sexual exploitation; CTQ = childhood trauma questionnaire; TLEQ = traumatic life events questionnaire.
